# Zinc binding of a Cys2His2-type zinc finger protein is enhanced by the interaction with DNA

**DOI:** 10.1007/s00775-023-01988-1

**Published:** 2023-02-23

**Authors:** Bálint Hajdu, Éva Hunyadi-Gulyás, Kohsuke Kato, Atsushi Kawaguchi, Kyosuke Nagata, Béla Gyurcsik

**Affiliations:** 1 Department of Inorganic and Analytical Chemistry, University of Szeged, Dóm Tér 7, 6720 Szeged, Hungary; 2 Laboratory of Proteomics Research, Biological Research Centre, Eötvös Loránd Research Network (ELKH), Temesvári Krt. 62, 6726 Szeged, Hungary; 3 Department of Infection Biology, Faculty of Medicine, University of Tsukuba, 1-1-1 Tennodai, Tsukuba, 305-8575 Japan

**Keywords:** Zinc finger protein, Zn(II)-affinity, DNA binding, Isothermal titration calorimetry, Electrophoretic mobility shift assay, Mass spectrometry

## Abstract

**Abstract:**

Zinc finger proteins specifically recognize DNA sequences and, therefore, play a crucial role in living organisms. In this study the Zn(II)-, and DNA-binding of 1MEY#, an artificial zinc finger protein consisting of three finger units was characterized by multiple methods. Fluorimetric, circular dichroism and isothermal calorimetric titrations were applied to determine the accurate stability constant of a zinc finger protein. Assuming that all three zinc finger subunits behave identically, the obtained thermodynamic data for the Zn(II) binding were *ΔH*_binding site_ =  − (23.5 − 28.0) kcal/mol (depending on the applied protonation state of the cysteines) and log*β*’_pH 7.4_ = 12.2 ± 0.1, being similar to those of the CP1 consensus zinc finger peptide. The specific DNA binding of the protein can be characterized by log*β*’_pH 7.4_ = 8.20 ± 0.08, which is comparable to the affinity of the natural zinc finger proteins (Sp1, WT1, TFIIIA) toward DNA. This value is ~ 1.9 log*β*’ unit higher than those determined for semi- or nonspecific DNA binding. Competitive circular dichroism and electrophoretic mobility shift measurements revealed that the conditional stability constant characteristic for Zn(II) binding of 1MEY# protein increased by 3.4 orders of magnitude in the presence of its target DNA sequence.

**Graphical abstract:**

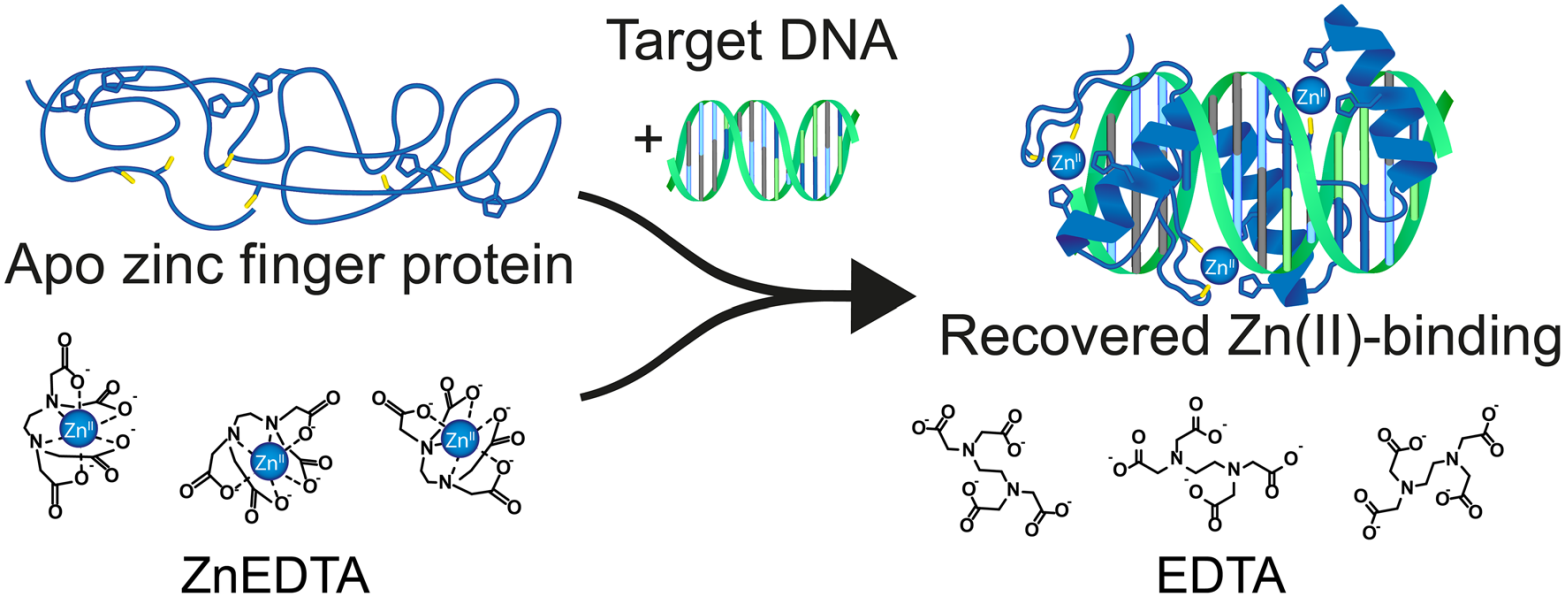

**Supplementary Information:**

The online version contains supplementary material available at 10.1007/s00775-023-01988-1.

## Introduction

Zinc finger proteins (ZFPs) are involved in DNA transcription, translation, error correction, metabolism, stimulus generation, cell division, and cell death by interacting with other proteins, small molecules, RNA or DNA in cells [1–6]. Usually zinc finger (ZF) motifs are responsible for the recognition of the target molecules, while the other protein domains for their actual function [7–13]. Commonly, the structure of a ZF motif is stabilized by the tetrahedral coordination of a Zn(II) ion and by the formation of a hydrophobic core [14]. The Cys2His2-type ZFs were first identified from *Xenopus laevis* by Aaron Klug’s research group in 1985 [3]. They form the most populous family of specific DNA recognition proteins [15]. The biotechnological significance of ZFs is given by the fact that a ZF unit recognizes and binds to three subsequent nucleotides in DNA, while several ZF units can be linked together to increase the specificity of the interaction. Furthermore, the DNA recognition of the ZF units can be reprogrammed. The designed ZF arrays were the first to be applied as DNA recognition domains fused to the FokI restriction endonuclease domain in artificial zinc finger nucleases (ZFNs) [16]. Since then, gene modification experiments are being performed with nucleases of this type further increasing their importance [17–23].

The Cys2His2 ZFPs can specifically bind DNA only in their Zn(II)-bound form. The coordination of Zn(II) to 2 cysteine and 2 histidine amino acid sidechains induces the protein folding into a characteristic ββα secondary structure. Therefore, their metal ion affinity is crucial in Zn(II) sequestering and proper functioning. Numerous studies have been addressed to investigate the coordination chemical and biophysical properties of ZFPs [11, 24–26], but to date there is a large deviation in the published metal-binding affinity data. The literature data on quantitative metal-binding properties of various single-unit ZF peptides were summarized [27] (Table S1), but there are very few data related to metal-binding properties of ZFPs larger than a single ZF unit (Table 1, vide infra). Furthermore, a limited number of studies on Zn(II)-binding of ZFPs bound to their molecular targets (*e.g.*, DNA) is published, although this may significantly modify their properties [28–31]. Furthermore, the improvement of the measurement methods over the years necessitates the reinvestigation of these systems [27]. The precise knowledge of the strength of the ZFP–Zn(II) interaction is also a prerequisite of understanding the effects of competitive toxic metal ions [32, 37].

**Table 1:** Average log*β’* values related to the interaction of various ZFPs with Zn(II); *RT* spectroscopic reverse titration, *CDc* competition with complexones monitored by circular dichroism spectroscopy, *cITC* competition with complexones monitored by ITC, *ED* equilibrium dialysis, *PAR* spectroscopic measurement of the competition with 4-(2-pyridylazo) resorcinol (PAR)

ZFP	Conditions	log*β*’ pH 7.4	Reference
1MEY# full	10 mM HEPES pH 7.4	12.2 (cITC)	Present work
		12.0 (CDc)	Present work
TFIIIA full	50 mM HEPES, pH 7.4, 50 mM KCl	8.0 (ED)	[34]
MTF1 full	100 mM HEPES, pH 7.0, 50 mM NaCl	11.3 (RT)*	[28]
	10 mM HEPES, pH 7.4, 100 mM NaClO_4_	9.1 (PAR)	[79]

*Recalculated to pH 7.4 by Kluska et al. [27]

Recently, we have purified a consensus peptide 1 (CP1)-based ZFP, 1MEY# by immobilized metal ion affinity chromatography followed by Ni(II) induced cleavage of the affinity tag [38]. This procedure yielded an amino-terminal Cu(II)/Ni(II) binding (ATCUN) motif at the N-terminus of the protein. The additionally bound metal ion within this motif posed a further challenge to determine the Zn(II)-affinity of the protein (for the details see Supplementary section S1, and Fig. S1). Here, we used fluorimetry, circular dichroism spectroscopy, isothermal calorimetric titration, mass spectrometry and electrophoretic mobility shift assay as independent methods to investigate the metal ion and DNA binding of the 1MEY# protein under various conditions.

## Experimental

### Materials

The construction of the genes as well as the expression and purification of the 1MEY# protein are detailed in Supplementary Experimental Sections S2 and S3. The procedures were monitored by tricine–sodium dodecyl sulfate–polyacrylamide gel electrophoresis (SDS PAGE) [39] using three-layered polyacrylamide gels. The bands were visualized by Coomassie Brilliant Blue staining, and Unstained Protein Molecular Weight Marker (Thermo Scientific) served as a reference.

### Mass spectrometric identification of the cleaved protein

Intact protein analysis was performed on an LTQ-Orbitrap Elite (Thermo Scientific) mass spectrometer coupled with a TriVersa NanoMate (Advion) chip-based electrospray ion source as described previously [40]. During top-down analysis *R* = 30,000 resolution was used at 400 m/z.

### Circular dichroism (CD) spectroscopy

CD spectra were recorded on a J-1500 Jasco spectrometer under constant nitrogen flow with a 20 nm/min scanning speed in the wavelength range of 180–330 nm. Synchrotron radiation (SR) CD spectra were recorded over the range of 170–330 nm at the CD1 beamline of the storage ring ASTRID at the Institute for Storage Ring Facilities (ISA), University of Aarhus, Denmark [31, 42].

All spectra were recorded with 1 nm steps and a dwell time of 2 s per step, using *l* = 0.1 or 0.2 mm quartz cells (SUPRA-SIL, Hellma GmbH, Germany). Each sample containing 10–20 µM protein was prepared separately in 10 mM 4-(2-hydroxyethyl)-1-piperazineethanesulfonic acid (HEPES) buffer (pH 7.4 or pH 8.2) and incubated at room temperature for 5 min prior measurement.

### Electrophoretic mobility shift assay (EMSA)

EMSA experiments were carried out as described previously [43] using 34 bp DNA probes including none (S0 DNA) or a single (S1 DNA) target sequence (5’-GAGGCAGAA-3’) of 1MEY#. The S0 DNA probe was obtained by hybridization of the Forward-S0: 5’-CTAGTTTGCTGAACTGGGGTCACATAGATTAATA-3’ and Reverse-S0: 5’-TATTAATCTATGTGACCCCAGTTCAGCAAACTAG-3’ oligonucleotides, while to construct the S1 DNA probe the Forward-S1: 5’-GAATTCCTGCTGAGAGGCAGAAACATAGGGGTCG-3’ and Reverse-S1: 5’-CGACCCCTATGTTTCTGCCTCTCAGCAGGAATTC-3’ oligonucleotides (the target sequence of 1MEY# is underlined) were hybridized. Oligonucleotides were obtained by solid phase synthesis (Invitrogen). FastRuler Ultra Low Range DNA Ladder (Thermo Scientific) was used as reference. The gels were stained in 0.5 ng/µl EtBr solution for 15 min and visualized by a Uvitec BTS 20MS gel documentation system.

### Isothermal titration calorimetry (ITC)

ITC experiments were carried out at 25 ± 0.1 °C in a Low Volume Nano ITC instrument (TA Instruments) in overfilled mode with stirring at 350 rpm. 50 µl of the titrant was injected at 0.5–2.5 µl aliquots (100–20 data points per titration) into 170 µl volume of the sample solution. 10 mM HEPES (pH 7.4) served as a working buffer, which was initially treated with 5 mg/dm^3^ Chelex® 100 cation exchange resin (Sigma-Aldrich) for 30 min at 25 °C, filtered through MF-Millipore 0.22 µm mixed cellulose ester membrane filter (Merck) and degassed. Protein samples were transferred into the working buffer using Amicon 3 K 0.5 ml filters (Merck) at 14,000×*g* at 15 °C for 6 × 5 min. This procedure yielded typically a protein solution of ~ 20 μM concentration, while the flow through during the last step of ultrafiltration served as background during the ITC titrations. The titrant was prepared by dilution of ethylenediaminetetraacetic acid (EDTA) stock solutions with the working buffer. The concentration of EDTA stock solution was determined complexometrically by titrating a known amount of Pb(II)-salt. Three parallel titrations were carried out. Between each protein–EDTA titration water–water and Ca(II)–EDTA reference systems were also measured. Sufficient waiting time (5–24 min) was applied between injections to allow the equilibrium to be reached (*i.e.,* to allow the signal to return to the baseline heat level). As the burette was immersed throughout the solution, diffusion of the titrant needed to be considered during the slow equilibrium process. This was achieved by the reference titrations, which showed a strong negative Pearson correlation (*r* =  − 0.715) between the measured heat and the injection interval time (Fig. S2). Therefore, heat corrections were applied if a measurement included longer than 5 min injection intervals. The heat effect of dilution was measured by titrating the corresponding protein flow through solution. These values were subtracted from the measured heat changes.

### ITC data evaluation

The Nano Analyze program (TA Instruments) includes various binding models. However, due to its limitations, it was only used for the evaluation of the water–water and Ca(II)–EDTA titrations, as well as to integrate the raw heat vs. time data sets. During the titrations of the holo-1MEY# protein with EDTA reactions (1–3) were supposed to occur:

1 $${\mathrm{Zn}}_{3}1\mathrm{MEY}\#+{\mathrm{EDTA}}^{*}={\mathrm{Zn}}_{2}1\mathrm{MEY}\#+{\mathrm{ZnEDTA}}^{*}$$

2 $${\mathrm{Zn}}_{2}1\mathrm{MEY}\#+{\mathrm{EDTA}}^{*}=\mathrm{Zn}1\mathrm{MEY}\#+{\mathrm{ZnEDTA}}^{*}$$

3 $$\mathrm{Zn}1\mathrm{MEY}\#+{\mathrm{EDTA}}^{*}={\mathrm{ZnEDTA}}^{*}+ 1\mathrm{MEY}\#$$

where ${\mathrm{EDTA}}^{*}$ represents the actual protonated state of EDTA under the measurement conditions. Assuming that the three ZF units behave identically and independently, the reaction can be simplified to Eq. (4), where $1\mathrm{MEY}\#\mathrm{^{\prime}}$ is a single ZF unit of 1MEY# ZFP.

4 $$\mathrm{Zn}1\mathrm{MEY}\#\mathrm{^{\prime}}+{\mathrm{EDTA}}^{*}={\mathrm{ZnEDTA}}^{*}+ 1\mathrm{MEY}\#\mathrm{^{\prime}}$$

Based on Eq. (4), a competition model can be used. The free concentrations can be calculated analytically, while the ΔH and log*β*’ values can be fitted using Solver add-in of Excel (Microsoft). Detailed derivations used in this work were described by Bent [44]. The equation describing the final heat change after every injection (${\Delta Q}_{i}$) has been modified to be applicable to the overfilled titration cell of NanoITC (see Eq. 5).

$${\Delta Q}_{i} =\left({V}_{0} \cdot {c}_{\mathrm{Zn}\left(\mathrm{II}\right),0,i} \cdot {x}_{\left[\mathrm{Zn}{1\mathrm{MEY}\#}^{\mathrm{^{\prime}}}\right],i} -\left({V}_{0} -{v}_{i}\right) \cdot {c}_{\mathrm{Zn}\left(\mathrm{II}\right),0,i-1} \cdot {x}_{\left[\mathrm{Zn}{1\mathrm{MEY}\#}^{\mathrm{^{\prime}}}\right],i-1}\right){\cdot \Delta H}_{\mathrm{ITC}}+$$

5 $$\left({V}_{0} \cdot {c}_{\mathrm{Zn}\left(\mathrm{II}\right),0,i} \cdot {x}_{\left[\mathrm{ZnEDTA}\right],i} -\left({V}_{0} -{v}_{i}\right) \cdot {c}_{\mathrm{Zn}\left(\mathrm{II}\right),0,i-1} \cdot {x}_{\left[\mathrm{ZnEDTA}\right],i-1}\right){\cdot \Delta H}_{\left[\mathrm{ZnEDTA}\right]}$$

where ${V}_{0}$ is the initial cell volume, ${v}_{i}$ is the *i*^th^ injection volume, ${c}_{\mathrm{Zn}\left(\mathrm{II}\right),0,i}$ is the total concentration of Zn(II) after the *i*^th^ injection, ${c}_{\mathrm{Zn}\left(\mathrm{II}\right),0,i-1}$ is the total concentration of Zn(II) after the $(i-1)$ ^th^ injection, ${x}_{\left[\mathrm{Zn}{1\mathrm{MEY}\#}^{\mathrm{^{\prime}}}\right],i}$ is the molar fraction of the Zn(II)-bound finger unit after the *i*^th^ injection, ${x}_{\left[\mathrm{ZnEDTA}\right],i}$ is the molar fraction of ZnEDTA^*^ complex after the *i*^th^ injection, ${\Delta H}_{\left[\mathrm{ZnEDTA}\right]}$ is the enthalpy change during the formation of ZnEDTA^*^ complex, which was obtained from the reference titrations and ${\Delta H}_{\mathrm{ITC}}$ is the overall enthalpy change during the Zn(II) complexation of the ZF unit. The calculation of the actual Zn(II) binding enthalpy of the ZF subunit (${\Delta H}_{\mathrm{Zn}1\mathrm{MEY}\#\mathrm{^{\prime}}}$) from the overall ${\Delta H}_{\mathrm{ITC}}$ enthalpy value can be found in the Supplementary Section S4.

### Fluorimetric measurements

2-[2-[2-[2-[bis(carboxylatomethyl)amino]-5-methoxyphenoxy]-ethoxy]-4-(2,7-difluoro-3-oxido-6-oxo-4a,9a-dihydroxanthen-9-yl)anilino]acetate (FluoZin-3), a Zn(II) selective fluorescent dye was applied to monitor Zn(II) release from 1MEY# in competition titrations by a CLARIOstar Plus plate reader (BMG Labtech). The absorption maximum of FluoZin-3 is at 494 nm, while it exhibits fluorescence at 516 nm when bound to Zn(II) with a pH independent stability constant of: log*β* = 8.16 [45]. The concentration of FluoZin-3 was determined spectrophotometrically (λ_max_ = 491 nm, ε_max_ = 71,143 M^–1^ cm^–1^, pH 7.4). FluoZin-3 samples (200 µl each) containing increasing amounts of holo-1MEY# or ZnCl_2_ (as reference) were separately assembled followed by 30 min incubation at 25 °C. Measurements were performed in 96 well polystyrene non-binding flat-bottom black microplates (Greiner Bio-One) at 25 °C using 480–490 nm excitation and 510–520 nm emission filters. The relative fluorescence of the holo-1MEY#–FluoZin-3 samples were calculated using the corresponding Zn(II)–FluoZin-3 value as a reference.

### Fluorescence anisotropy

Fluorescence anisotropy measurements were performed by CLARIOstar Plus plate reader (BMG Labtech). 474–490 nm excitation and 510–550 nm emission filters were applied to monitor the fluorescence of 6-carboxyfluorescein (FAM) in 200 µl DNA–protein samples in 96 well polystyrene non-binding flat-bottom black microplates (Greiner Bio-One). The 28 bp labelled double-strand DNA probe containing two 1MEY# target sequences (underlined) was assembled by hybridization of the Forward-S1: 5’-FAM-CCGAGGCAGAATTCGTTCTGCCTCAG-3’, fluorescein-labelled and Reverse-S1: 5’-TAMRA-CTGAGGCAGAACGAATTCTGCCTCGG-3’, tetramethylrhodamine-labelled oligonucleotides. Oligonucleotides were obtained by solid phase synthesis (Invitrogen).

## Results and discussion

### Zn(II) binding of 1MEY# ZFP

**Fig. 1:**
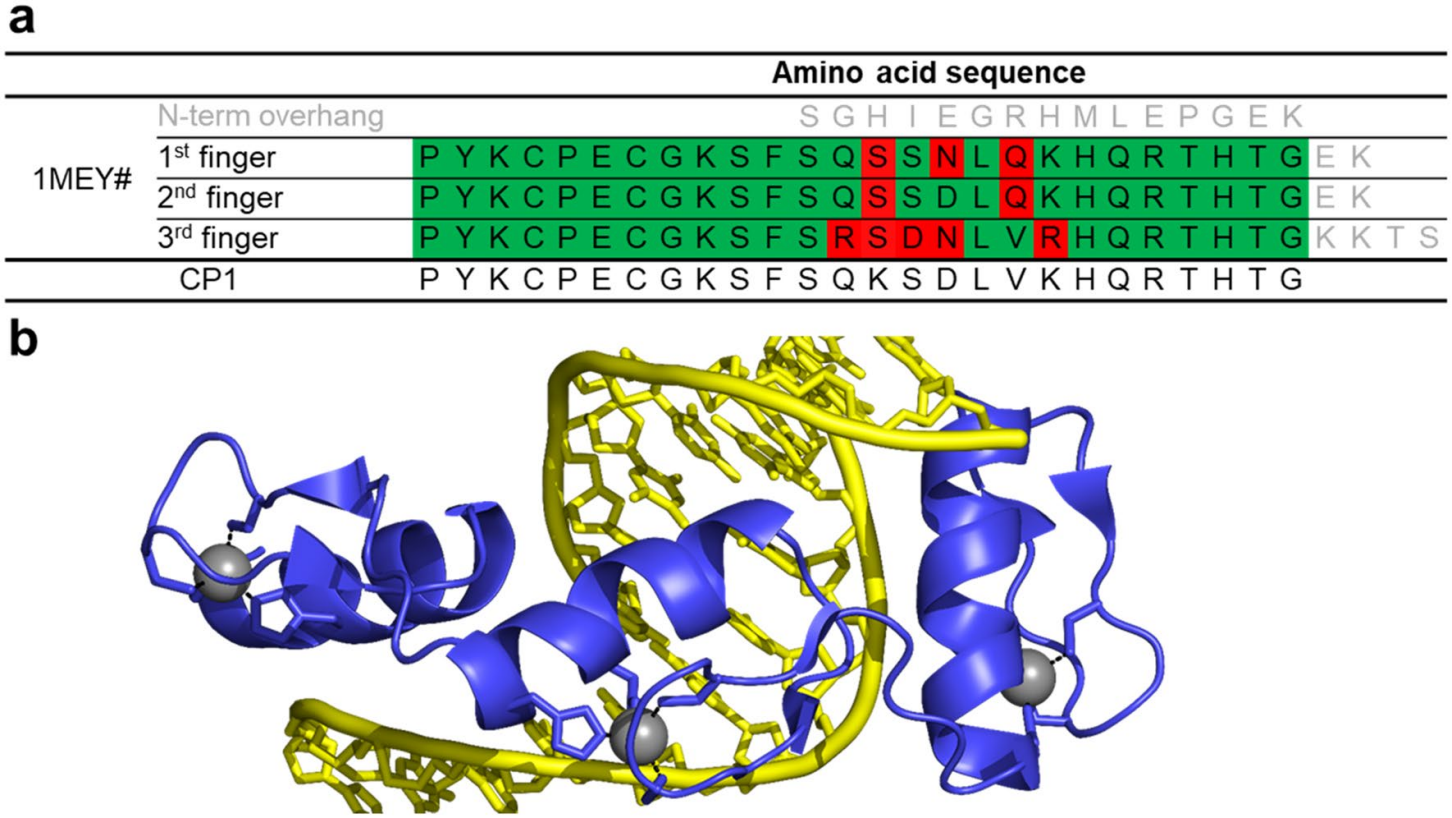
**a** Alignment of the amino acid sequence of 1MEY# ZFP (derived from 1MEY ZFP [61]) with CP1, the 26 amino acid long consensus Cys2His2 model peptide established and investigated by Berg et al. [62]. The differences observed in the DNA recognition region (highlighted by red background) presumably do not affect the metal ion binding by the conserved cysteine and histidine sidechains. Green background indicates identity with CP1. **b** Cartoon representation of crystal structure of the CP1-based ZFP in complex with DNA. 1MEY# is a modified version of this protein [38]. ZFP: blue, Zn(II): grey sphere, DNA: yellow (PyMOL representation of 1MEY PDB [61])

A new purification strategy of the 1MEY# protein (Fig. 1) was applied in the present work as described in Supplementary Sections S2 and S3, as well as in Fig. S3. The protein was purified by immobilized Ni(II)-affinity chromatography similar to the previously described procedure [39], but instead of the Ni(II)-promoted hydrolysis the affinity tag was cleaved off by the specific ULP1 protease [46]. The SDS PAGE images shown in Fig. S4 demonstrated the success of the protein purification. As a result, we expected to avoid the metalation of the ATCUN motif formed upon ULP1 cleavage. ESI–MS measurements (Fig. S5) supported the SDS–PAGE results concerning the purity of the preparation. The deconvoluted experimental monoisotopic mass (11,479.6 Da) for acidified protein solution was in agreement with the calculated value for the apo-1MEY# MH^+^ ion (11,479.5 Da). On the other hand, a Ni(II) ion was detected in holo-1MEY# beside the three Zn(II) ions (experimental: 11,726.2 Da *vs.* calculated: 11,726.2 Da). This demonstrated the high capability of the ATCUN motif to bind Ni(II) ions. Thus, 1MEY# could acquire Ni(II) from the Ni(II)–NTA resin during the purification procedure. Nevertheless, this is in line with the stabilities of the Ni(II) complexes of XXH-type peptides as models being in the range of (log*β* = 19.19–23.37) [47–57] compared to that of the Ni(NTA) complex log*β* = 10.75–11.54 [58, 59]. Furthermore, the metal complexes of the ATCUN motif are kinetically inert (33% of Ni(II) was still complexed in ATCUN even after treatment with 1 (v/v)% formic acid for 10 min) [38, 60] and it took ~ 400 h to completely remove it by 66 eqs EDTA at 25 °C (Fig. S1 b,). Therefore, in most cases we did not attempt to remove Ni(II) and used the holoprotein in its purified form in the further experiments, except in case of ITC measurements, where Ni(II)-free proteins were investigated (for the details of the Ni(II) removal procedure, see supplementary section S1). The circular dichroism spectrum of the purified 1MEY# protein was similar to that of 1MEY# previously purified by Ni(II)-induced hydrolysis [38] suggesting the identity of the secondary structure compositions of the two products (Fig. S6).

Investigation of the Zn(II) binding affinity of ZFPs needs consideration of several limiting factors, especially in direct experiments. Visible absorption spectrometry is not applicable due to the closed d-shell of Zn(II), while in the UV range of the thiolate to Zn(II) charge transfer band [63] the absorbance is strongly affected by e.g., any change in the buffer, ionic strength, eventual oxidation of the tiol groups. The concentration range is limited by the Zn(II) contamination of the environment that makes the measurements ambiguous at low concentrations, while Zn(OH)_2_ precipitate forms above pH 7.4 in the mM range. Furthermore, the Zn(II)-free ZFPs are unstable, aggregate easily, and their cysteine residues are sensitive to oxidation. Most of these difficulties may be overcomed by starting the experiments with the holoprotein [64]. Depending on the buffer conditions, precipitation of the protein may occur above 20 μM concentration. Competitors shall be used if the apparent cumulative stability constant is higher than 10^9^; however, the time to reach the equilibrium might be long [27, 65]. Only a few quantitative studies were published about the Zn(II) binding of ZFPs constructed from more than one ZF subunit, and the determined values were greatly dependent on the conditions and methods applied (see Table 1).

FluoZin-3, a selective Zn(II)-sensing fluorescent probe [45] was applied as a competitor to determine the apparent stability constant of the Zn1MEY# binding sites. However, the fluorimetric titration results shown in Fig. S7 indicated that FluoZin-3 could not be an effective competitor for Zn(II). From these data only an rough estimate on the Zn(II) affinity of 1MEY# was obtained. Accordingly, *β*’_Zn1MEY# bs pH=7.4_ should be larger than 10^9^. Thus, a stronger chelator than FluoZin-3 was needed for further experiments. In the lack of such commercially available fluorescent probe, CD spectroscopy was an obvious choice to follow the collapse of the characteristic ββα secondary structure of the holo-1MEY# ZFP upon removal of the Zn(II) ions by a non-chiral competitor. The results of the titrations with EDTA (log*β*_ZnEDTA_ = 16.5) suggested a quantitative reaction (Fig. S8), which was useful to monitor the metalation status of the protein, but did not allow the calculation of the affinity constant. As a weaker chelator, ethylene glycol-bis(β-aminoethyl ether)-N,N,N′,N′-tetraacetic acid (EGTA) seemed to be applicable (log*β*_ZnEGTA_ = 14.5), but the equilibrium was extremely slow with this competitor. Even 600-fold EGTA excess was insufficient to obtain the CD spectrum similar to that of completely unfolded 1MEY# (Fig. S9) within 1 h incubation time.

**Fig. 2:**
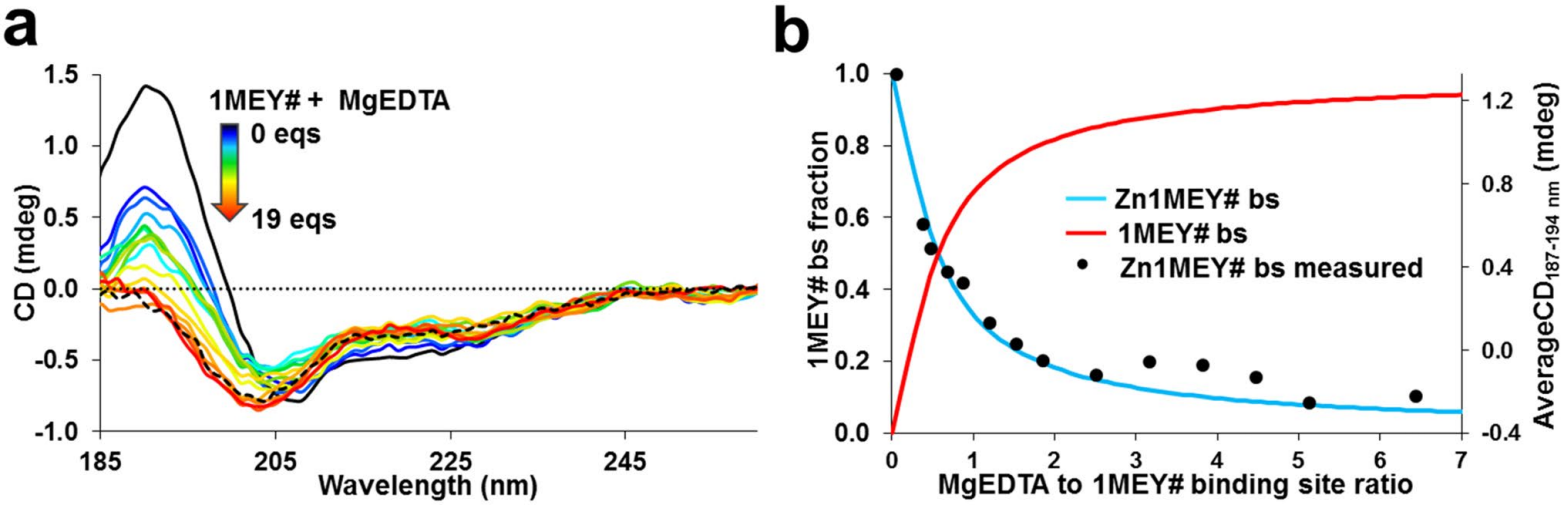
**a** Series of the CD spectra of 1MEY# recorded in the presence of increasing amounts of MgEDTA complex. Black dashed line: 1MEY# in the presence of 5 eqs EDTA (1.67 eqs to 1MEY# binding site) after 5 min incubation (c(holo-1MEY#) = 7 µM in 10 mM HEPES (pH 7.4); *l* = 0.1 mm) Reaction mixtures were prepared separately and were incubated for 12 h at 25 °C. **b** Species distribution diagram showing the partition of 1MEY# binding site calculated from the ellipticity values between the 187–194 nm range by the PSEQUAD program (full lines) [66]. Measured average ellipticity values between 187 and 194 nm range (black dots) are presented for comparison. 1MEY# bs notation refers to the 1MEY# binding site

As an alternative, 1MEY# was titrated with the solution of the MgEDTA complex. The competition of Mg(II) and Zn(II) for EDTA occurred (Fig. 2a) within 30 min at 6.5 eqs of MgEDTA. Yet the separately assembled samples were incubated for 12 h to assure that the equilibrium was reached. Assuming three identical Cys2His2 Zn(II)-binding sites in 1MEY# the species distribution diagram was calculated and shown in Fig. 2b. The apparent stability constant log*β*’_pH 7.4_ = 12.0 ± 0.1 was obtained from the evaluation of the CD titration data using the PSEQUAD program.

Isothermal calorimetric titrations were also carried out to confirm the above stability by an independent method. It has been previously suggested that competitive ITC measurements with a chelator might be suitable for studying high-stability ZF motifs [27, 65]. The Zn(II) binding of metallothioneins was investigated [67], but to the best of our knowledge, this is the first time to determine the thermodynamics of a ZFP complex by this method.

**Fig. 3:**
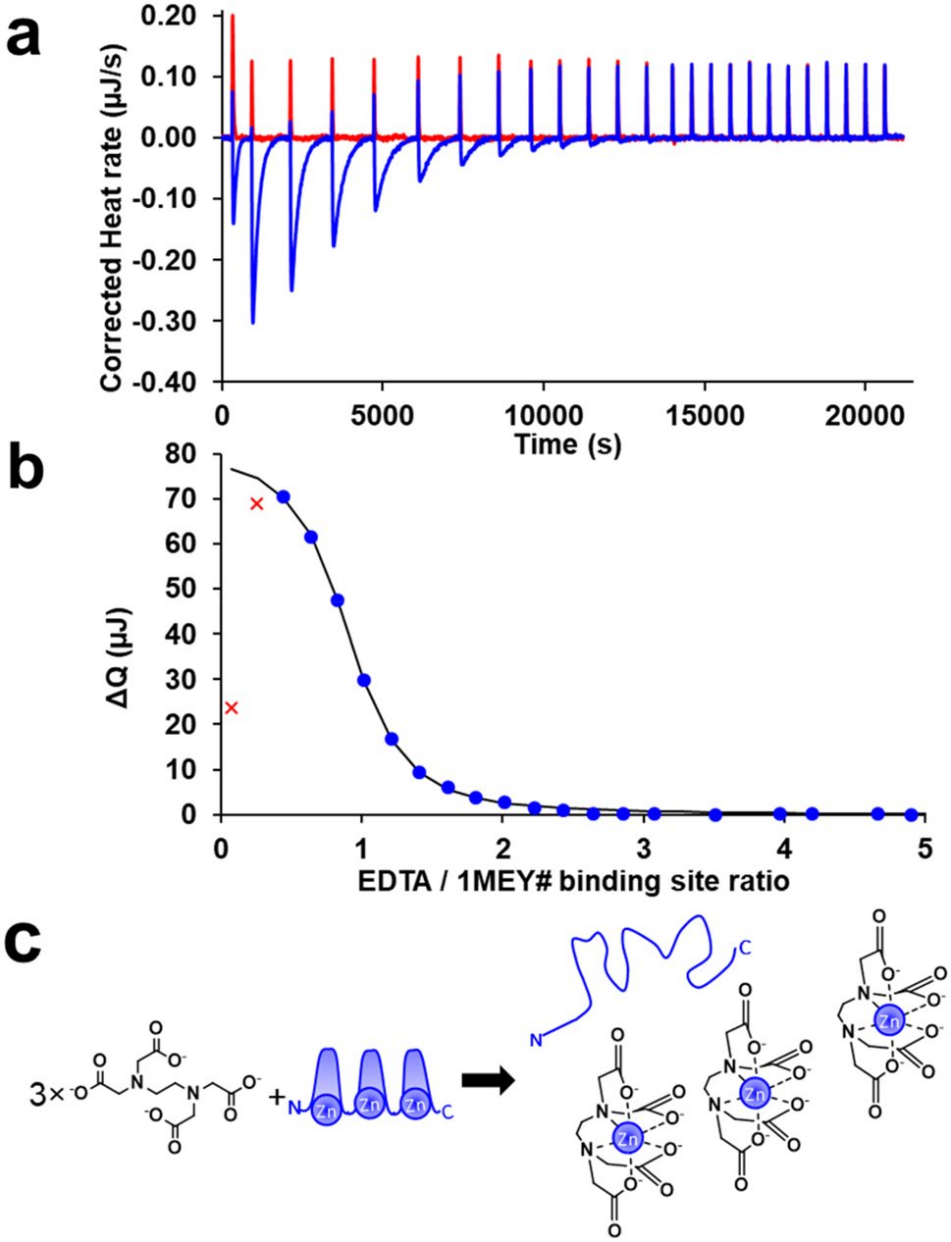
**a** Representative ITC curves of 10 µM holo-1MEY# ZFP (blue line), or 1MEY# flow through (red line) titrated by 2 µl 500 µM EDTA aliquots (10 mM HEPES (pH 7.4)); **b** integrated background-corrected heat changes during 1MEY#–EDTA titration (blue dots), and the fitted heat change (black line); The very first experimental points (symbolized by red crosses) were avoided from the evaluation process. **c** Schematic representation of the competition process (for simplicity, the protonation of EDTA is not indicated)

The sensitivity of the sigmoidal curve of ITC close to the equivalence point allowed for titration of the holo-1MEY# with EDTA. Competitive ITC has several advantages: environmental Zn(II)-contamination, Zn(OH)_2_ precipitation, and cysteine oxidation do not occur to a measurable extent and, therefore, do not interfere with the measurement (Supplementary Section S5 and Fig. S10), while low volumes (≥ 190 µl) and concentrations (≥ 10 µM) can be used. The only real limiting factor may be slow kinetics. While the CD spectra revealed that the equilibrium was established rapidly in the 1MEY#–EDTA system, 30 min intervals between the injections (Fig. 3a) were necessary in the ITC experiments close to the equivalence point instead of a typical 5 min ITC interval. The published data on the kinetics of the competition reaction are rather diverse in the literature. Sénèque and Latour reported that the equilibrium in the Zn(II):CP1:EDTA 1:1:1 system can be reached only in 250 min at pH 7.35 (log*β*’_ZnCP1_ _pH 7.4_ = 15.7) [65]. Heinz et al. found three-orders of magnitude faster exchange kinetics using the CP1-Δ8 peptide, in which a glycine was deleted at 8^th^ position (log*β*’_ZnCP1-Δ8 pH 7.4_ = 11.4) [65, 68], indicating that small changes in the amino acid sequence may greatly affect the competition rate. Generally, the aforementioned CP1-like ZFs with higher thermodynamic stability tend to exert slower kinetics.

Based on the similarity of the amino acid sequences of all three subunits of 1MEY# with that of the CP1 model ZF peptide (Fig. 1) similar apparent stability constants were expected for the individual ZF subunits. Therefore, the integrated ITC titration curves were fitted with a competition model considering three separate identical Zn(II) binding sites of 1MEY#. The good fit of the sigmoidal pattern of the 1MEY# titration curve supported the above hypothesis (Fig. 3b). The evaluation of the ITC data yielded average results corresponding to a single ZF subunit. As the first step, the ZnEDTA reaction enthalpy was determined separately to be Δ*H*_ZnEDTA_ = –17.24 kJ/mol = –4.1 kcal/mol, which is in a good agreement with the literature values ranging from − 14.98 to − 23.5 kJ/mol [69–73] (for the details of the calculations see Supplementary Sections S4). This and the log*β*’_pH 7.4_ = 13.56 stability value [74] was applied for ZnEDTA formation in the subsequent fitting procedure. Previously, the protonation of ~ 0.5 eqs cysteine per ZF subunits was suggested [75]. Using this value in the evaluation process, the calculated Δ*H*_binding site_ =  − 23.5 ± 1.3 kcal/mol enthalpy value for Zn(II) binding of a ZF unit was similar to the values reported by Blasie and coworkers for the CP1 model peptide (Δ*H*_CP1_ =  − 22.9 ± 1.1 kcal/mol [76]; Δ*H*_CP1_ =  − 23.4 ± 1.0 kcal/mol [75] in 200 mM PIPES (pH 7.0), 50 mM NaCl buffer). The enthalpy determined in HEPES buffer was slightly different (ΔH_CP1_ =  − 27.6 ± 0.6 kcal/mol; 200 mM HEPES (pH 7.0), 50 mM NaCl) [75]. Very recently, Kluska et al. published a new investigation of CP1 model peptide variants, where the p*K*_a_ values of the thiols in the peptide were determined to be p*K*_a1_^SH^ = 7.77; p*K*_a2_^SH^ = 9.15 [77], corresponding to an average protonation of 1.68 equivalents of cysteines per ZF subunit under the measurement conditions [77]. Applying this value in the calculations, Δ*H*_binding site_ =  − 28.0 ± 1.4 kcal/mol enthalpy could be obtained, which is by 5.1–5.3 kcal/mol larger than the values determined for the K/S mutant of CP1 peptide (–22.9 kcal/mol [76]; –22.68 kcal/mol [77]), while it is much closer to the enthalpy of the initial CP1 in HEPES buffer. The determined enthalpy values independently of the number of protonated cysteines are within the range of the values determined for the CP1 peptide variants over the years. Based on this, it cannot be claimed, that the linker sequences and the terminal overhangs would affect significantly the thermodynamics of Zn(II) binding.

The log*β’*_pH 7.4_ = 12.2 ± 0.1 obtained for the Zn1MEY# ZF unit is close to the value determined from the MgEDTA competition experiments by CD. The log*β’*_pH 7.4_ values determined for Zn(II)–CP1 system over the years vary between 12.0 and 15.7 depending on the measurement method and buffer conditions (Table S1). The Zn(II)-affinity of 1MEY# is almost identical to the low-end stability constant value determined for the CP1 model peptide, [62, 78] (Table S1), while 3.5 units lower than the most recently determined log*β’* value [65]. The CP1-derived ZFP binds Zn(II) with a similar affinity to the model peptides of naturally occurring ZF subunits (Table S1) and to full ZFPs, such as TFIIIA and MTF1 (Table 1).

**Fig. 4:**
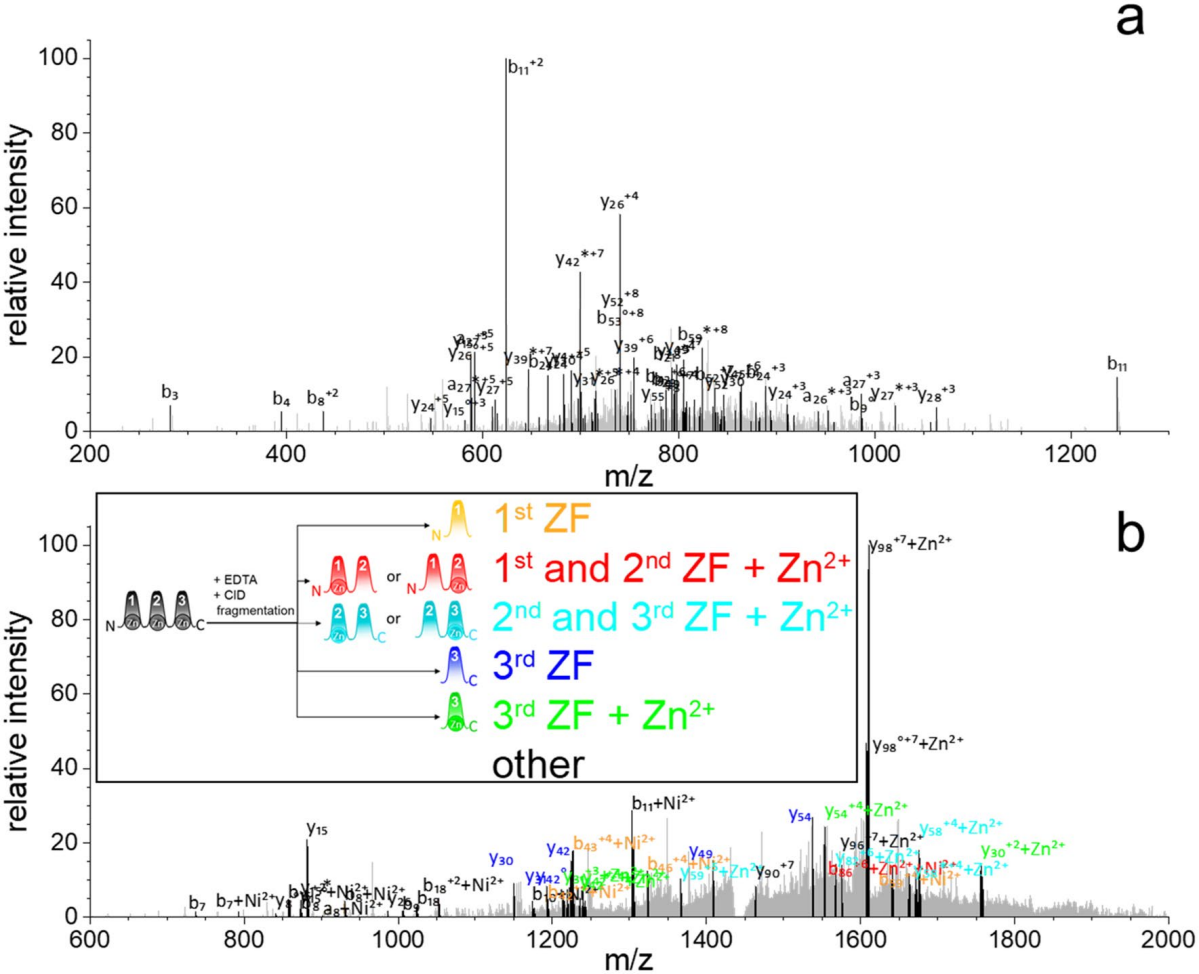
**a** MS/MS analysis of apo-1MEY#. The precursor with m/z = 766 (*z* = 15) was selected for CID fragmentation. **b** MS/MS analysis of holo-1MEY# in the presence of 12.5 eqs EDTA. The precursor with m/z = 1451 (*z* = 8) corresponding to 1MEY# coordinated by a Zn(II) and a Ni(II) ion was selected for CID fragmentation. Labels of 1MEY# fragments are color-coded according to the ZF subunits: yellow: first ZF; blue: third ZF; green: third ZF with a Zn(II); red: first and second ZF with a coordinated Zn(II); light blue: second and third ZF binding one Zn(II), black: other fragments. The presence of Ni(II) was observed in the N-terminal fragments due to the ATCUN motif. NH_3_ losses are represented by *, H_2_O losses by °. Assigned peaks are black, unassigned are grey

Mass spectrometric measurements suggested that there is no ZF subunit with paramount Zn(II) binding capacity in 1MEY# ZFP. Metal binding of 1MEY#, however, significantly reduced the protein fragmentation rate and modified protein charge state under MS conditions (Fig. S11a). Fragmentation of the entire holoprotein was not feasible (data not shown), while the apoprotein yielded well-defined fragment peaks under the same measurement conditions (Fig. 4a) (Table S2). The fragmentation of Zn_1_1MEY# ZFP species could be achieved in the presence of 12.5 eqs EDTA. Under these conditions only a few free protein or two Zn(II)-containing protein was detected (Fig. S11b). The major fragmentation products lost only a few N- or C-terminal amino acids, while Zn(II) remained bound. Fragments in which Zn(II) coordinated to the third ZF, the third or second, or the first or second ZF units have been identified by comparing the higher signal to noise ratio peaks with simulations (Fig. 4) (Table S3).

### DNA binding of 1MEY# ZFP

**Fig. 5:**
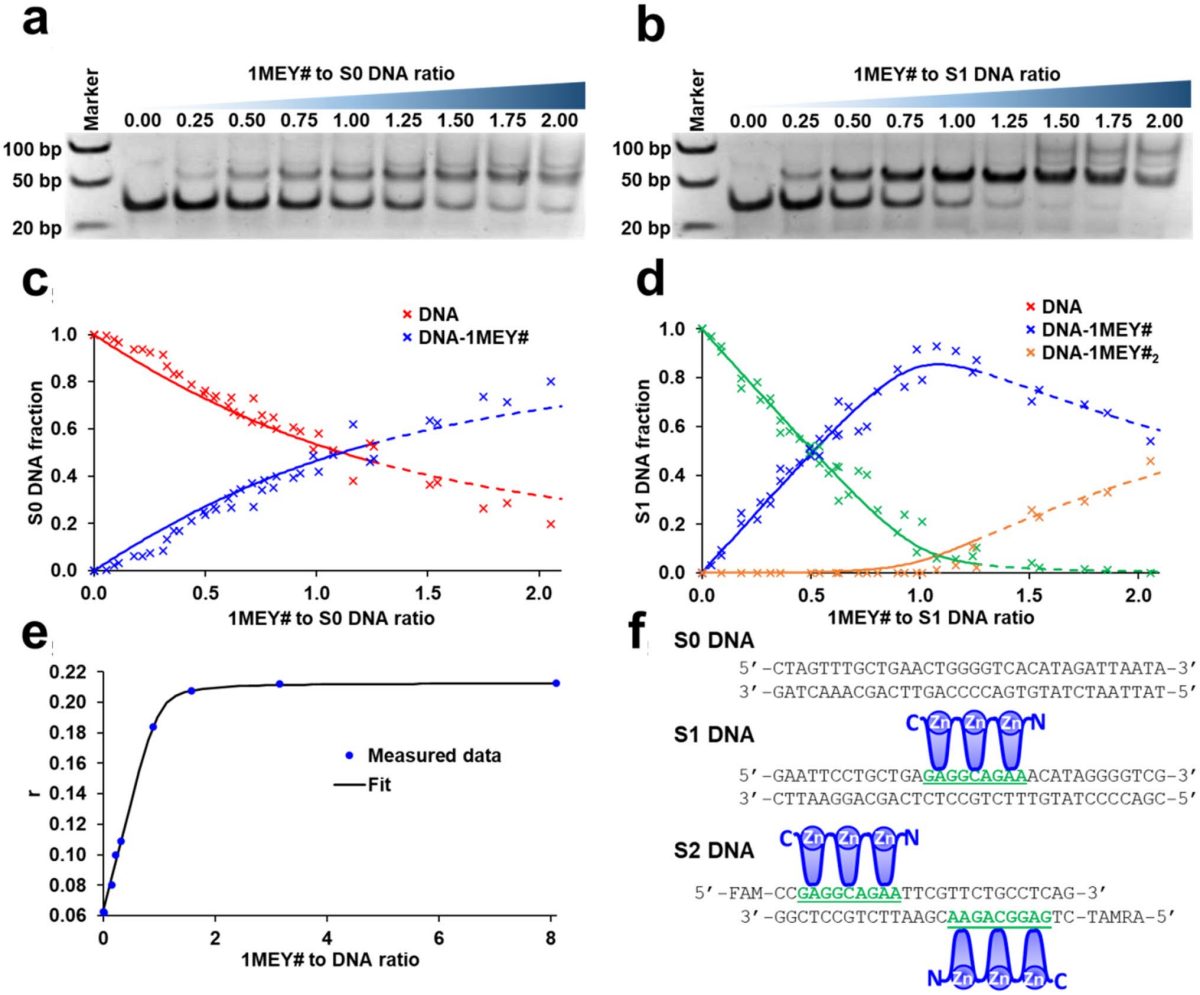
Representative electrophoretic mobility shift assays of **a**, nonspecific S0 DNA and **b**, specific S1 DNA in the presence of increasing amounts of 1MEY# ZFP (c(DNA) = 0.88 µM, 10 mM HEPES, 150 mM NaClO_4_, 10 m/v % glycerol buffer (pH 7.4)). Marker: FastRuler Ultra Low Range DNA Ladder (Thermo Scientific). **c** Distribution of S0 DNA or **d**, S1 DNA among various DNA–ZFP complexes in the presence of increasing amounts of 1MEY# ZFP. DNA fractions (separate points) were calculated based on the intensities of four independent experiments. Band intensities were analyzed by ImageJ [81]. **e** Fluorescence anisotropy of specific S2 labelled DNA (with 2 ZFP recognition site) in the presence of increasing equivalents of 1MEY# ZFP (c(DNA-binding-site) = 0.4 µM, 10 mM HEPES, buffer (pH 7.4)). 200 µl samples were separately assembled and incubated for 15 min at 25 °C and then loaded into the plate. Calculations (solid lines) were performed by PSEQUAD. Dashed lines indicate, that the simulation has higher uncertainty, since multiple bands appear on the gel, which are hard to quantify. **f** Sequence of S0, S1 and S2 DNA containing 0, 1 and 2 ZFP binding site. 5’-GAGGCAGAA-3′ 1MEY# ZFP binding site (green, underlined)

The ZFP published in ref. [61] (Protein Data Bank code: 1MEY) recognizes the 5’-G(A/G)G(G/T)C(A/G)GAA-3’ DNA sequence. Since it was cocrystallized with the 5’-GAGGCAGAA-3’ DNA this was accepted as the main target sequence of 1MEY and 1MEY#, as well [38] but no DNA binding affinity was determined for this particular protein so far. Based on the quantitative evaluation of the electrophoretic gel mobility shift experiments (Fig. 5) the interaction of 1MEY# with specific DNA was found ~ 1.9 log*β’* unit stronger than that with the nonspecific one (Table 2). The holo-ZFP binds nonspecific S0 DNA with log*β’* = 6.27 ± 0.02 and by increasing protein excess additional faint band appeared but the quantification of the intensity of this band was uncertain. Therefore, we could not calculate a reliable affinity value from it (Fig. 5a, c). Enhanced bandshift was observed with the 34 bp S1 DNA containing the underlined specific sequence (Fig. 5b, d, f) and the 1:1 protein–DNA complex was characterized by log*β’* = 8.20 ± 0.08. This value was also supported by the results of the fluorescence anisotropy experiments (Fig. 5e) (Table 2). The above results are in good agreement with the literature data on the selectivity of designed ZFPs. The log*K* values increased to a similar extent when the nonspecific DNA was exchanged to a specific target sequence [80].

**Table 2:** Log*β*’ values related to the interaction of ZFPs with their specific or nonspecific DNA counterparts

	ZF	Conditions	SDNA:Prot	NSDNA:Prot	Method	Ref
			1:1	1:1		
1MEY#	3	10 mM HEPES, pH 7.4, 150 mM NaClO_4_, 10 m/v % Glycerol	8.20 ± 0.08	6.27 ± 0.02	EMSA	Present work
1MEY#	3	10 mM HEPES, pH 7.4	8.0		ANI	Present work
Sp1	3	25 mM Tris, pH 8.0, 100 mM KCl, 10 m/v % Glycerol 2 mM DTT, 50 µg/ml BSA, 2 µg/ml dI–dC	6.9^a^ 7.6^b^		EMSA^r^	[83]
		10 mM Tris, pH 8.0, 50 mM NaCl, 100 µM ZnCl_2_, 1 mM β-Mercaptoethanol, 0.05 v/v % NP-40, 5 v/v % Glycerol	7.4^b^		EMSA^r^	[84]
Sp1 1–2	2	10 mM Tris, pH 8.0, 50 mM NaCl, 100 µM ZnCl_2_, 1 mM β-Mercaptoethanol, 0.05 v/v % NP-40, 5 v/v % Glycerol	6^b^		EMSA^r^	[84]
Sp1 2–3	2	10 mM Tris, pH 8.0, 50 mM NaCl, 100 µM ZnCl_2_, 1 mM β-Mercaptoethanol, 0.05 v/v % NP-40, 5 v/v % Glycerol	6.8^b^		EMSA^r^	[84]
Sp1C	3	25 mM Tris, pH 8.0, 100 mM KCl, 10 m/v % Glycerol 2 mM DTT, 50 µg/ml BSA, 2 µg/ml dI–dC	7.3^a^ 8.4^b^		EMSA^r^	[83]
WT1	4	20 mM Tris, pH 7.5, 150 mM KCl, 1 mM MgCl_2_, 1 mM DTT, 1 mg/ml CM-Dextran, 0.005 v/v % Surfactant P20	8.2		SPR	[85]
	4	20 mM Tris, pH 7.5, 100 mM KCl, 5 Mm MgCl_2_, 1 mM DTT, 5 µM ZnCl_2_, 5 µg/ml dI-dC, 100 µg/ml BSA	8.94		FBA^r^	[86]
WT1 1–3	3	20 mM Tris, pH 7.5, 150 mM KCl, 1 mM MgCl_2_, 1 mM DTT, 1 mg/ml CM-Dextran, 0.005 v/v % Surfactant P20	7.75		SPR	[85]
WT1 2–4	3	20 mM Tris, pH 7.5, 150 mM KCl, 1 mM MgCl_2_, 1 mM DTT, 1 mg/ml CM-Dextran, 0.005 v/v % Surfactant P20	8.37		SPR	[85]
WT1 2–3	2	20 mM Tris, pH 7.5, 150 mM KCl, 1 mM MgCl_2_, 1 mM DTT, 1 mg/ml CM-Dextran, 0.005 v/v % Surfactant P20	6.65		SPR	[85]
MTF-1	6	40 mM MOPS, pH 7.0, 20 mM NaCl	8.58^c^ 8.04^d^		ANI	[87]
EGR1	3	20 mM Tris, pH 7.5, 100 mM KCl, 5 Mm MgCl_2_, 1 mM DTT, 5 µM ZnCl_2_, 5 µg/ml dI-dC, 100 µg/ml BSA	8.45		FBA^r^	[86]
		10 mM Tris, pH 7.5, 0.2 µM ZnCl_2_, 150 mM KCl	6.9		ANI	[88]
			8.2		ANI	[89]
Zif268	3	15 mM HEPES, pH 7.8, 50 mM KCl, 50 mM K-Acetate, 50 mM K-Glutamate, 5 mM MgCl_2_, 20 µM ZnSO_4_, 100 µg/ml BSA, 5 v/v% Glycerol, 0.1 w/v % NP-40	10.6		EMSA^r^	[90]
TFIIIA full	9	20 mM HEPES, pH 7.5, 50 mM KCl, 1 mM MgCl_2_, 5 mM DTT, 50 µM ZnCl_2_, 12 v/v % Glycerol	5.34^e^		EMSA^r^	[91]
TFIIIA 1–3	3	20 mM HEPES, pH 7.5, 50 mM KCl, 1 mM MgCl_2_, 5 mM DTT 50 µM ZnCl_2_, 12 v/v % Glycerol	5.75^e^		EMSA^r^	[91]
			6.16^f^		EMSA^r^	[91]
			6.54^ g^		EMSA^r^	[91]
		50 mM K-phosphate, pH 6.67, 100 mM NaCl, 50 µM ZnCl_2_	6.88		FLU	[92]
			6.88		ITC	[92]
YY1	4	20 mM HEPES, pH 7.5, 150 mM NaCl, 5 mM MgCl_2_, 100 µM Zn(Ac)_2_, 1 mM TCEP	6.25		ITC	[93]
		25 mM Tris, pH 8.5, 100 mM NaCl, 10 mM MgCl_2_, 5 mM DTT, 100 µM ZnCl_2_, 0.02 w/v % NaN_3_, 100 µg/ml BSA, 0.04 w/v % PEG-20000	6.78	4.44	ANI	[94]
		10 mM Tris, pH 7.9, 100 mM NaCl, 10 mM MgCl_2_, 5 mM DTT, 100 µM ZnCl_2_, 0.05 v/v % Surfactant P20	7.36		SPR	[94]

The binding constants for 1MEY# were calculated from the data shown in Fig. 5 by PSEQUAD. Literature stability values were recalculated to log*β*’. *SDNA* Specific DNA, *NSDNA* nonspecific DNA, *TFIIIA full* native X*enopus laevis* transcription factor, *TFIIIA 1–3* first three ZF sections of TFIIIA, *EGR1 or ZIF268* Early growth response protein 1, *WT1* Wilms Tumor Protein, *Sp1C* Sp1 protein, where peptide backbone is altered to match consensus peptide-1, *MTF-1* Metal regulatory transcription factor 1. DNA binding sites of 1MEY#: 5’-GAGGCAGAA-3’; WT1: 5’-GCGTGGGCGTGT-3’; EGR1 and ZIF268: 5’-GCGTGGGCG-3’; TFIIIA 1–3: 5’-GGATGGGAG-3’; MTF-1: 5’-GAGCTCTGCACTCCGCCCGAAAA-3’. *EMSA* electromobility gelshift assay, *ANI* Fluorescence anisotropy measurement, *SPR* Surface Plasmon Resonance measurement, *FBA* Filter Binding assay, *FLU* Fluorimetric measurement, *ITC* Isothermal Titration Calorimetry.^a^ 5’-GAGGCGGGG-3’ DNA probe was used

^b^5’-GGGGCGGGG-3’ DNA probe was used

^c^All MTF-1 ZF units loaded with Zn(II)

^d^Only the 4 strong Zn(II)-binding motif is loaded

^e^72 bp probe probe was used

^f^21 bp probe probe was used

^g^13 bp probe probe was used

^r^log*β*’ value has high uncertainty, since < 1 pmol DNA amounts were used with radiolabeled detection

Since the 34 bp long S1 DNA contains a 25 bp guanine-rich sequence in addition to the 9 bp target site, 1MEY# can also interact semi-specifically [82], i.e., by finding a partial recognition site, or nonspecifically with these DNA sections. Therefore, additional band evolved around 100 bp which can be characterized by log*β*_2_’ = 14.26 ± 0.10 stability value. By subtracting the log*K*_1_ value dedicated for the specific binding, a log*K*_2_ = 6.06 value can be obtained, which is close to the nonspecific DNA-affinity; therefore, it can be concluded, that the additional binding is nonspecific (Fig. 5a, b, c, d).

Despite the different measurement conditions, the DNA binding affinity of 1MEY# is comparable or slightly higher to that of MTF-1—if that protein only binds four Zn(II)-ions and, therefore, recognizes a 12 bp sequence—and the Zn(II) binding affinity of 1MEY# is also comparable or slightly higher compared to MTF-1, as well (Table 1). On the other hand, TFIIIA with significantly weaker Zn(II) binding, has lower affinity for DNA probes. This might suggest a correlation between the Zn(II) and DNA binding ability of the ZFPs.

### 1MEY#-DNA competition with EDTA

**Fig. 6:**
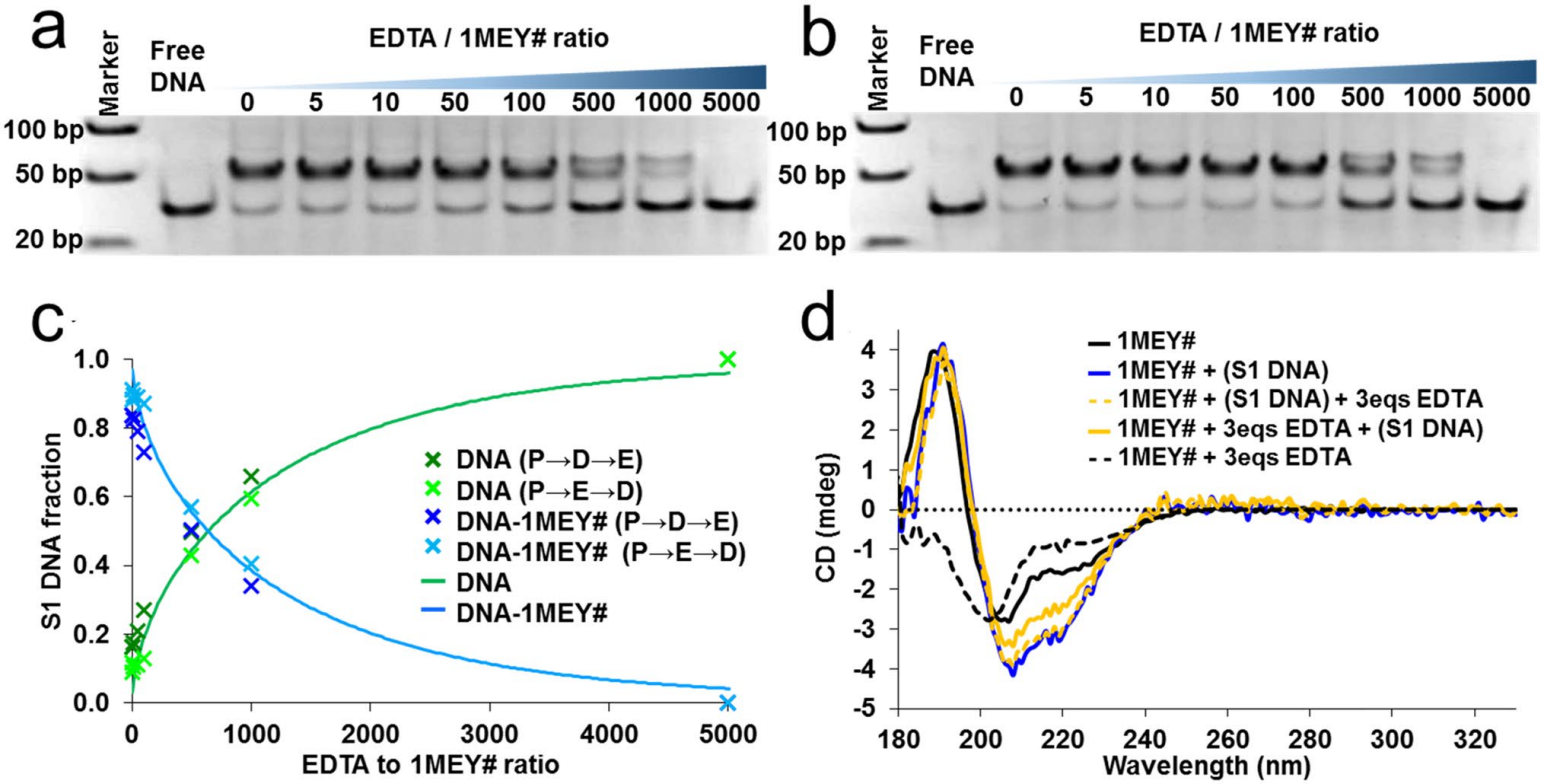
**a** Electrophoretic gel mobility shift assay of 1MEY# with specific S1 DNA in the presence of increasing equivalents of EDTA. 1 μM 1MEY# ZFP, 1 μM S1 DNA in 10 mM HEPES, 150 mM NaClO_4_ buffer (pH 7.4); **b** electrophoretic gel mobility shift assay of 1MEY# in the presence of increasing equivalents of EDTA. After 1 h incubation 1 eq specific S1 DNA was added to the samples. 1 μM 1MEY# ZFP, 1 μM S1 DNA in 10 mM HEPES, 150 mM NaClO_4_ buffer (pH 7.4). **c** Distribution of S1-DNA among the free and protein-bound forms as calculated from the band intensities of the electrophoretic gel mobility shift assay image; (P → D → E) represents the assembly order in which first the ZFP–DNA complex was assembled, then increasing amount of EDTA was added to the samples, while in case of (P → E → D), first, the ZFP was treated with EDTA for 1 h, then S1 DNA was added to the solution. Full lines: simulated distribution performed by PSEQUAD (Supplementary Section S6). **d** Comparison of the CD spectra of the holo-1MEY# ZFP, in the presence and absence of 3 eqs EDTA, and S1 DNA. The CD spectrum of S1 DNA was subtracted from the relevant spectra. Protein concentrations were normalized to 18.8 µM. 10 mM HEPES (pH 7.4) *l* = 0.1 mm

The DNA binding of ZFPs may affect their interaction with Zn(II). Addition of EDTA excess to 1MEY# in the presence of specific DNA did not change the CD spectrum significantly (dashed yellow spectrum, Fig. 6d). Treating the DNA-free holo-1MEY# with the same amount of EDTA for the same time caused the collapse of the secondary structure (dashed black spectrum). Interestingly, the CD spectrum indicated the recovery of the ββα-like secondary structure upon mixing the EDTA treated unfolded protein sample with the specific S1-DNA (full yellow spectrum, Fig. 6d). This suggests, that the addition of the specific DNA template to apo-1MEY# could promote the uptake of Zn(II) ions from EDTA. Similar result was obtained in the EMSA titration experiments. Gel mobility shift was observed regardless of the order of sample assembly (Fig. 6a, b). DNA could only be completely liberated by increasing the EDTA excess to ~ 5000 fold (5 mM). By overlapping the quantified gel intensities of the samples assembled in different order (Protein → DNA → EDTA and Protein → EDTA → DNA), a good agreement of the data was observed (Fig. 6c, separate points). The data could be simulated with the smallest error using log*β’*_pH 7.4_ = 15.6 ± 0.15 value as the conditional Zn(II)-affinity of 1MEY# ZFP (Supplementary Section S6 and Fig. 6c, full lines), while in the absence of DNA, this value used to be log*β’*_pH 7.4_ = 12.2 ± 0.1 (Table 1).

Previously, it was reported that 0.5 mM EDTA abolished the DNA–MTF-1 interaction within 1 h [28, 29]; however, whole cell extracts were used in both cases, thus the exact ratio of EDTA to protein is unknown. A more significant effect was visible with Sp1, which has provoked the interest of researchers over the years. Petering et al. investigated the Zn(II)–Sp1–DNA system in the presence of EDTA and other chelators concluding that the ZFP–DNA complex is either kinetically inert or thermodynamically stable, but using ~ 500 eqs EDTA excess the interaction could be ceased [30, 31]. Only electrophoretic mobility shift assay was applied in these studies. Therefore, it was not clear whether the band shift in the experiments with a protein → EDTA → DNA sample assembly order occurred, because EDTA could not remove all Zn(II) from ZFP in a given timeframe (kinetic aspect), or because the ZFP was able to recover Zn(II) from EDTA in the presence of DNA (thermodynamic aspect). The latter phenomenon is difficult to confirm with Sp1, since its DNA binding affinity is not outstanding. In contrast, the modification of the peptide backbone of the Sp1 protein to obtain the consensus peptide sequence, resulting Sp1C protein lead to much stronger DNA binding. If interpreted correctly, the observed band shift with the reaction mixture assembled in protein → EDTA → DNA order was hypothesized to occur, because EDTA was unable to remove Zn(II) from the protein for both kinetic and thermodynamic reasons [83]. Here, we demonstrated by applying CD and EMSA as independent methods that EDTA was indeed not able to remove Zn(II) from 1MEY# in the presence of DNA, furthermore, the apo-protein could recover Zn(II) from the ZnEDTA complex in the presence of specific DNA. Thus, the interaction with DNA increased the conditional Zn(II) binding affinity of 1MEY# by 3.4 log units. Such stabilization or recovery of the holoprotein structure and function occurs most probably with other ZFPs binding tightly to their cognate DNA targets.

## Conclusions

It is known, that Cys2His2 ZFPs are only able to recognize their DNA target sequence in their appropriate ββα configuration, and Zn(II) is essential for the formation of such structure. As a result, Zn(II) binding plays a key role in biological function of ZFPs. Numerous competitor ligands inside the cell may affect the interaction between Zn(II) and ZFPs influencing their structure and function. The quantitative evaluation of the experimental data on Zn(II) and DNA binding of 1MEY#, a CP1-derived three finger ZFP suggested that the protein binds both the metal ion and DNA strongly, and that the presence of the specific DNA target may significantly increase the apparent Zn(II) affinity in a cooperative manner. This provides a favourable condition to perform their function in the cellular environment including strong competitor molecules. Our findings are in good agreement with the qualitative electrophoretic gel mobility shift data in the literature, suggesting similar behaviour of the Sp1 ZFP in the presence of EDTA, N,N,N′,N′-tetrakis(2-pyridinylmethyl)-1,2-ethanediamine, glutathione, 4-(2-pyridylazo)resorcinol and metallothionein [29, 31, 33, 83] competitors. Nevertheless, toxic metal ions could also compete with Zn(II) for the ZFP binding sites. Recently, we reported that DNA could not protect 1MEY# against Ag(I) attack [43]. Further studies are needed to fully understand the mechanism of these complex processes.

## Supplementary Information

Below is the link to the electronic supplementary material.

Supplementary file1 (PDF 1147 kb)

## Data Availability

The datasets generated and analyzed during the current study are available from the corresponding author upon reasonable request.
