# Supplementary material for: Zinc binding of a Cys2His2-type zinc finger protein is enhanced by the interaction with DNA
Source: J Biol Inorg Chem. 2023 Feb 23;28(3):301–15. doi: 10.1007/s00775-023-01988-1 (PMC10036435; doi:10.1007/s00775-023-01988-1)
Supplement: Supplementary file 1 — Supplementary file1 (PDF 1147 kb) [file 775_2023_1988_MOESM1_ESM.pdf]

ELECTRONIC SUPPLEMENTARY INFORMATION

Zinc binding of a Cys2His2 type zinc finger protein is enhanced by the interaction with DNA

Bálint Hajdu[a], Éva Hunyadi-Gulyás[b], Kohsuke Kato[c], Atsushi Kawaguchi[c], Kyosuke Nagata[c], Béla Gyurcsik[a]*

[a]*Department of Inorganic and Analytical Chemistry, University of Szeged, Dóm tér 7, H-6720 Szeged, Hungary*

[b]*Laboratory of Proteomics Research, Biological Research Centre, Eötvös Loránd Research Network (ELKH), Temesvári krt. 62, H-6726 Szeged, Hungary*

[c]*Department of Infection Biology, Faculty of Medicine, University of Tsukuba, 1-1-1 Tennodai, Tsukuba 305-8575, Japan*

*Corresponding author

e-mail: gyurcsik@chem.u-szeged.hu

EXPERIMENTAL

Section S1. Characterization of the N-terminal Ni(II)-ATCUN complex

The effect of the N-terminally bound Ni(II) was investigated using UV-Vis absorption spectrometry. Low intensity peaks dedicated to the Ni(II)-ATCUN complex were observed in the Vis wavelength range around 420 nm [1], and the 280 nm absorbance value was increased by ~20% as well, due to the N-terminal complex (Fig. S1 a).

The kinetics of Ni(II)-removal by EDTA from the Ni(II)-ATCUN complex was extremely slow, it took around 2 weeks to reach the endpoint in the presence of 66 eqs EDTA at 25 °C which suggested, that this metal complex wouldn't interfere with the investigation of the Zn(II)-binding of ZF units (Fig. S1 b).

This measurement was strengthened by CD-followed EDTA titration of holo-1MEY# ZFP, where instant decrease of the 190 nm positive peak was visible resulting a rather sharp breakpoint around 3.2 eqs EDTA (Fig. S8). If Ni(II) had been removed by EDTA first, no change would be visible during the titration up to one equivalent, and if Ni(II) and Zn(II) were removed at the same time, we would have a break point at 4 equivalents.

Despite these results, the titrations with (Mg)-EDTA followed by ITC and CD were carried out with Ni(II)-free 1MEY# ZFP protein, as well. Removal of Ni(II) could be achieved by >500 eqs EDTA during 50 °C 5 hour incubation in the presence of ~100 eqs TCEP in 10 mM 4-(2-hydroxyethyl)-1-piperazineethanesulfonic acid (HEPES, pH 7.4) buffer. Then, the EDTA concentration was decreased below 1 μM by buffer exchange to 100 μM TCEP, 10 mM HEPES (pH 7.4) buffer using Amicon 3K 15 ml filters (Merck) at 14000× g, 4×5 min, 15 °C. Thereafter, 500 μM Zn(II) (final concentration) was added to the sample, which was followed by buffer exchange to 10 mM HEPES (pH 7.4) as described previously. The successful removal of Ni(II) was confirmed by UV-Vis measurements.

Section S2. Gene construction

The 1MEY# zinc finger protein (ZFP) described earlier in ref. [2] was expressed using a pETM11-SUMO3 vector, which was kindly provided by Dr. Milan Kožíšek (IOCB Prague, Proteases of Human Pathogens Research Group). The ZFP gene was recloned from the original pET-16b-P-1MEY# plasmid into the new vector by standard cloning procedures. The 303 bp long P-1MEY# gene was amplified in polymerase chain reaction (PCR) by DreamTaq polymerase (Thermo Scientific) using the 5'-aaaaggatcCGGCCATATCGAAGGTC-3' forward and 5'-ttttctcgagTCCTTAAGAGGTTTTTTACCAG-3' reverse primers allowing for BamHI and XhoI recognition sites (underlined) in the amplified gene at its 5' and 3' termini, respectively (Fig. S3). The resulted DNA fragments were double digested by BamHI and XhoI restriction endonucleases (Thermo Scientific) and treated by FastAP thermosensitive alkaline phosphatase (Thermo Scientific). In parallel, the pETM11-SUMO3 plasmid was also digested by the same enzymes. The digested DNA mixtures were purified by extraction with phenol-chloroform mixture and a subsequent precipitation by ethanol [3]. The insert and the vector were mixed in ~ 100:1 molar ratio and ligated by T4 ligase (Thermo Scientific). *E. coli* DH5α cells were transformed, plated and cultured in 5 mL LB medium. The resulting DNA was purified using EZ-10 Spin Column Plasmid DNA Miniprep Kit (BioBasic). The success of the mutagenesis was verified by standard DNA sequencing procedure.

The pETM11-SUMO3 system introduced an N-terminal hexa-His-SUMO3 tag instead of the previously applied deca-His tag [2]. This allowed the protein to be purified by Ni(II)-affinity chromatography in the same way as before, but now the affinity tag could be removed not only by the previously described Ni(II)-promoted hydrolysis [2], but also by the ULP1 protease which selectively cleaves the sequence after two glycines following the SUMO3 domain [4]. The applied ULP1 protease itself also has a hexa-His affinity tag so it can be purified with Ni(II)-affinity chromatography.

Section S3. Protein expression and purification

The 1MEY# protein and the ULP1 protease was expressed in *E. coli* BL21 (DE3) cells, cultured to OD_{600} ~ 1.0 at 37 °C for 8 h, when the protein expression was induced by IPTG (isopropyl β-D-1-thiogalactopyranoside) at 0.2 mM final concentration, at 25 °C for 8 h. Cells were cultured in standard Lysogeny Broth (LB) medium [5] containing either ampicillin (100 μg/ml final concentration) or kanamycin (50 μg/ml final concentration). Cells were harvested by centrifugation at 4000× g for 15 min at 4 °C, yielding ~ 10 g wet cells / 1 L of the bacterial culture. 10 ml 1× binding buffer (500 mM NaCl; 100 mM HEPES (pH 8.2); 5 mM imidazole; 0.1 v/v % Triton X-100) was used to resuspend 1 g of the cell pellet. Cells were then lysed by ultrasonication at 50% amplitude (10 × 30 s) using a VCX 130 PB (130 W) ultrasonic processor equipped with a 129 mm long titanium probe with a 13 mm tip diameter. The extract was centrifuged at 4000× g for 35 min at 4 °C.

The soluble fraction of the lysate was mixed with 1/20× bed volume of Ni(II)-loaded His•Bind resin (Novagen) preequilibrated with 1× binding buffer and then equilibrated for additional 1 h at 4 °C. The resin was washed three times with 2× bed volume 1× wash buffer1 (500 mM NaCl; 100 mM HEPES (pH 8.2); 50 mM imidazole), and 3 times with 2× bed volume 1× wash buffer2 (150 mM NaCl; 100 mM HEPES (pH 8.2); 60 mM imidazole). At this point the resin portions loaded with 1MEY# and ULP1 were mixed together. $Zn(ClO_4)_2$ was added to the suspension at 50 μM final concentration followed by rotation at 16 °C for 12 h to cleave the hexa-His-SUMO3 affinity tag. Zn(II)-excess was applied to make it sure that the protein remained in its holo-form during the whole procedure and thus, to protect it from the oxidation at cysteine residues. After this incubation, 100 mM HEPES (pH 7.4) was added to the resin to reach final imidazole concentration of ~ 20 mM. The rotation was continued for 1 h at 16 °C and then the supernatant containing pure 1MEY# protein was separated from the resin together with the resin-bound hexa-His-SUMO3 affinity tag and ULP1 protease. The above purification steps were monitored by SDS PAGE (Fig. S4). The buffer of the purified 1MEY# sample was exchanged to 10 mM Cl^--free HEPES (pH 7.4) using Amicon 3K 15 ml filters (Merck) at 4000× g, 8×30 min at 15 °C followed by filtration through a 0.22 μm, Ø = 13 mm PES filter (Merck).

Section S4. ITC enthalpy evaluation

Section S4.1. Evaluation of the Zn(II) – EDTA reference ITC titrations

As it was indicated in the manuscript, the enthalpy of the Zn(II)–EDTA binding process was determined experimentally under the same conditions (10 mM HEPES (pH 7.4)) applied in the competition reaction. $ZnCl_2$ was titrated with EDTA in the same buffer mentioned above. EDTA to buffer titrations were performed to obtain the dilution heat in HEPES buffer and this was subtracted from the Zn(II)–EDTA titration to obtain the baseline corrected enthalpy ($\Delta H_{ITC} = -16.2$ kJ/mol).

Using the pK_a values of 2.07; 2.75; 6.26 and 10.34 [6], the protonation state of EDTA was simulated by PSEQUAD [7] at pH = 7.4. Accordingly, 93.4% is in H_1EDTA, 6.5% in H_2EDTA and 0.1% in non-protonated form. Thus, upon Zn(II)-complexation 1.064 equivalents of protons are released (charge states are not indicated):

$$\mathrm{Zn(II)} + 0.934\ \mathrm{H_1EDTA} + 0.065\ \mathrm{H_2EDTA} + 0.001\ \mathrm{EDTA} = \mathrm{ZnEDTA} + 1.064\ \mathrm{H^+} \quad \text{(s1)}$$

These protons react with HEPES:

$$\mathrm{H^+} + \mathrm{HEPES} = \mathrm{H_1HEPES} \quad \text{(s2)}$$

Protonation enthalpy values for EDTA ($\Delta H_{K_1} = -22.3$ kJ/mol; $\Delta H_{K_1} = -17.2$ kJ/mol) [8], and for HEPES ($\Delta H_{H_1HEPES} = -21.01$ kJ/mol) [9] at 25 °C was taken from literature. The following equation was solved to obtain the enthalpy of the Zn(II)–EDTA interaction:

$$\Delta H_{\mathrm{ZnEDTA}} = \Delta H_{\mathrm{ITC}} - \Delta \mathrm{n}_{H^+} \cdot \Delta H_{\mathrm{H_1HEPES}} - \Delta \mathrm{n}_{\mathrm{H_1EDTA}} \cdot \Delta H_{K_1} - \Delta \mathrm{n}_{\mathrm{H_2EDTA}} \cdot \left(\Delta H_{K_1} + \Delta H_{K_2}\right) \quad \text{(s3)}$$

Section S4.2. Evaluation of the holo 1MEY# – EDTA competition ITC titration

The refined $\Delta H_{\mathrm{ZnEDTA}}$ enthalpy, was used to fit the competition titration data. EDTA to 1MEY# flow through[1] titrations were carried out to obtain the dilution heat, which was subtracted from the heat of the competition titration. The following equation was used for data fitting:

$$\Delta Q_i = \Delta \mathrm{n}_{\mathrm{Zn1MEY\#',}i} \cdot \Delta H_{\mathrm{ITC}} + \Delta \mathrm{n}_{\mathrm{ZnEDTA,}i} \cdot \Delta H_{\mathrm{ZnEDTA}} \quad \text{(s4)}$$

where $\Delta H_{\mathrm{ZnEDTA}}$ referred to the previously determined enthalpy for the ZnEDTA complex formation and ΔH_{ITC} to all other processes in the system. From the fitted ΔH_{ITC} the reaction

[1] Flow through was obtained during the ultrafiltration of 1MEY# ZFP to the measurement buffer (10 mM HEPES pH = 7.4) using Amicon 3K ultra filters as indicated in Section 2.5 in the manuscript.

enthalpy of Zn(II) binding to the ZF subunits of 1MEY# was calculated based on the following equation:

$$\Delta H_{\mathrm{Zn1MEY\#'}} = \Delta H_{\mathrm{ITC}} + \Delta \mathrm{n}_{\mathrm{H_1EDTA}} \cdot \Delta H_{K_1} + \Delta \mathrm{n}_{\mathrm{H_2EDTA}} \cdot (\Delta H_{K_1} + \Delta H_{K_2}) + \Delta \mathrm{n}_{H^+} \cdot \Delta H_{\mathrm{H_1HEPES}} + \Delta \mathrm{n}_{\mathrm{H_1Cys}} \cdot \Delta H_{\mathrm{H_1Cys}} \quad \text{(s5)}$$

where $\Delta H_{\mathrm{Zn1MEY\#'}}$ is the enthalpy of the Zn(II)-binding to a single subunit of 1MEY# and the enthalpy of cysteine protonation is: $\Delta H_{\mathrm{H_1Cys}} = -29.7\ \mathrm{kJ/mol}$ [10]. In this process, 1.064 equivalents of protons dissociate from EDTA and beside the buffer, the free cysteine thiolates of 1MEY# can be protonated as well. In theory at pH 7.4 it would be expected, that the two cysteines takes up two protons, but Blasie et al., found experimentally that for the CP1 model peptide only ~0.5 cysteine per ZF subunit was protonated probably due to the positively charged sidechains close to cysteines [11]. Due to the high sequence similarity between CP1 and the ZF units of 1MEY# (Fig. 1 a) this result was accepted in the fitting procedure. The remaining protons were supposed to interact with HEPES. Thus, the following equation was solved:

$$\Delta H_{\mathrm{Zn1MEY\#'}} = \Delta H_{\mathrm{ITC}} - 0.934 \cdot \Delta H_{K_1} - 0.065 \cdot (\Delta H_{K_1} + \Delta H_{K_2}) + 0.564 \cdot \Delta H_{\mathrm{H_1HEPES}} + 0.5 \cdot \Delta H_{\mathrm{H_1Cys}} \quad \text{(s6)}$$

Section S5. Effect of EDTA treatment on cysteine oxidation

The ellipticity decrease cannot be addressed to cysteine oxidation, because as it was proved in earlier research, cysteine oxidation cannot occur while Zn(II) is coordinated, and after it is removed, it does not matter whether the free cysteines get oxidized. It is also demonstrated by other researchers, that the chelation reactions with ZFs can be slow [12].

Holo-ZFPs with high Zn(II)-affinity are not sensitive to Cys oxidation due to the protecting effect of Zn(II) [13, 14]. After Zn(II) removal (e.g. with EDTA) cysteines might become more sensitive therefore, it was investigated to which extent cysteines are oxidized during EDTA treatment if no anaerobic conditions are applied, but the samples are sealed from air to model the ITC reaction cell conditions. No detectable Cys oxidation occurred during EDTA treatment of 1MEY# during 60 minutes at room temperature in 10 mM HEPES (pH 7.4). under these conditions during the incubation time based on DTNB assay (Fig. S10).

Section S6. Simulation of zinc finger Zn(II)-affinity in the presence of DNA

The determined Zn(II)-affinity ($\log\beta'_{pH = 7.4} = 12.2 \pm 0.1$)[2] of 1MEY# ZFP refers to the average Zn(II)-binding of one ZF subunit inside the protein if all subunits have identical Zn(II)-affinity.

If the protein's DNA binding and Zn(II) binding should be simulated at the same time during EDTA treatment, the following equations can be written:

$$\mathrm{DNA(Zn_3 1MEY\#)_1 \rightleftharpoons DNA + Zn_3 1MEY\#} \quad \text{(s7)}$$

$$\mathrm{Zn_3 1MEY\# + EDTA^* \rightleftharpoons Zn_2 1MEY\# + ZnEDTA^*} \quad \text{(s8)}$$

$$\mathrm{Zn_2 1MEY\# + EDTA^* \rightleftharpoons Zn1MEY\# + ZnEDTA^*} \quad \text{(s9)}$$

$$\mathrm{Zn1MEY\# + EDTA^* \rightleftharpoons ZnEDTA^* + 1MEY\#} \quad \text{(s10)}$$

where EDTA* represents the actual protonated state of EDTA under the measurement conditions.

In this case the subunits of 1MEY# cannot be treated identically, statistical considerations must be taken into account during the estimation of $K'_{\mathrm{Zn_1 1MEY\#}}$, $K'_{\mathrm{Zn_2 1MEY\#}}$ and $K'_{\mathrm{Zn_3 1MEY\#}}$.

J. Bjerrum introduced the following equation to estimate the stepwise stability constants of a system:

$$\frac{K_j}{K_{j+1}} = f_j x^2 \quad \text{(s11)}$$

where x is the “spreading factor” and f_j is the value of the quotient of the constants to be expected on a statistical basis [11, 12]. In this case the connection between the average determined stability constant $\overline{K}$ and the stepwise stability constants K_j can be written as:

$$K_j = \frac{(N-j+1)}{j}\overline{K} \cdot x^{(N+1-2j)} \quad \text{(s12)}$$

where j is the number of occupied binding sites, N is the total number of identical binding sites. If the binding sites are identical it can be assumed, that $x = 1$, thus in case of 1MEY# the following equations can be written:

$$K'_{\mathrm{Zn_1 1MEY\#}} = \frac{(3-1+1)}{1}\overline{K} \cdot 1^{(3+1-2\cdot1)} = 3\overline{K} \quad \text{(s13)}$$

$$K'_{\mathrm{Zn_2 1MEY\#}} = \frac{(3-2+1)}{2}\overline{K} \cdot 1^{(3+1-2\cdot2)} = \overline{K} \quad \text{(s14)}$$

$$K'_{\mathrm{Zn_3 1MEY\#}} = \frac{(3-3+1)}{3}\overline{K} \cdot 1^{(3+1-2\cdot3)} = \frac{\overline{K}}{3} \quad \text{(s15)}$$

[2] In this chapter it will be labeled as $log\overline{K}$ for simplicity.

TABLES

Table S1. logβ' values related to the interaction of various ZF units with Zn(II) as summarized by Kluska et al. [14]. DT: spectroscopic direct titration; RT: spectroscopic reverse titration; PAR: spectroscopic measurement of the competition with PAR; CDc: competition with complexones monitored by circular dichroism spectroscopy; ITC: isothermal titration calorimetry; rITC: reverse isothermal titration calorimetry; Pot: potentiometry; TRT: spectroscopic three-step reverse titration.

ZF	Conditions	logβ' pH 7.4	Reference
CP1	100 mM HEPES, pH 7.0, 50 mM NaCl	12.5 (RT)*	[17]
		12.0 (RT)*	[18]
	50 mM MOPS, pH 7.0, 100 mM KCl	15.7 (CDc)*	[12]
	20 mM Tris, pH 7.4, 100 mM NaCl	14.49 (CDc)	[19]
CP1-Δ8	50 mM MOPS, pH 7.0, 100 mM KCl	11.4 (CDc)*	[12]
CP1 (2015)	20 mM Tris, pH 7.4, 100 mM NaCl	12.3 (CDc)	[19]
CP1 K/S	20 mM Tris, pH 7.4, 100 mM NaCl	14.0 (CDc)	[19]
TFIIIA-2	20 mM HEPES, pH 7.0, 50 mM NaCl	9.4 (RT)*	[20]
MTF1-1	50 mM HEPES, pH 7.0, 100 mM $NaClO_4$	12.4 (CDc)*	[21]
	50 mM HEPES, pH 7.4, 100 mM $NaClO_4$	11.4 (PAR)	[22]
	100 mM HEPES, pH 7.0, 50 mM NaCl	11.3 (RT)	[23]
	10 mM HEPES, pH 7.4, 100 mM $NaClO_4$	8.9 (PAR)	[24]
MTF1-2	10 mM HEPES, pH 7.4, 100 mM $NaClO_4$	9.9 (PAR)	[24]
MTF1-3	10 mM HEPES, pH 7.4, 100 mM $NaClO_4$	9.3 (PAR)	[24]
MTF1-4	10 mM HEPES, pH 7.4, 100 mM $NaClO_4$	9.4 (PAR)	[24]
MTF1-5	10 mM HEPES, pH 7.4, 100 mM $NaClO_4$	9.8 (PAR)	[24]
MTF1-6	10 mM HEPES, pH 7.4, 100 mM $NaClO_4$	9.3 (PAR)	[24]
WT1-3	50 mM HEPES-HCl, pH 6.5	10.5 (ITC)*	[25]
WT1-4	20 mM MES, pH 5.25, 100 mM KCl	8.9 (DT)*	[26]
Sp1-3	50 mM HEPES, pH 7.4, 100 mM $NaClO_4$	12.7 (CDc)	[21]
	50 mM HEPES, pH 7.0, 100 mM NaCl	10.0 (RT)*	[27]
	500 mM Tris, pH 7.4	7.6 (ITC)	[28]
		8.2 (rITC)	[28]
	50 mM Tris, pH 7.0	10.4 (RT)*	[28]
Zn-F10	50 mM HEPES, pH 6.5	8.3 (ITC)*	[25]
ZF133-11	50 mM HEPES, pH 7.4, 100 mM $NaClO_4$	12.5 (CDc)	[21]
	100 mM KNO_3	12.5 (Pot)	[21]
ZF278-1	50 mM HEPES, pH 7.4, 100 mM NaClO4	13.0 (TRT)	[21]

*Recalculated to pH 7.4 by Kluska et al. [14].

Table S2. Assigned fragments of 766 m/z precursor after the MS/MS analysis of apo 1MEY#. Assignment was performed using Protein Prospector

ATCUN		C-term	1st ZF		3rd ZF	1st and 2nd ZF	2nd and 3rd ZF
b_{3}	a_{10}	y_{8}-H_2O	b_{40}	a_{40}	y_{27}	a_{74}-NH_3	y_{55}
b_{4}	a_{11}	y_{8}-NH_3	b_{42}	a_{44}-NH_3	y_{27}-H_2O		y_{73}
b_{5}	a_{14}	y_{15}	b_{42}-H_2O	a_{45}-NH_3	y_{27}-NH_3		
b_{8}	a_{15}-NH_3	y_{15}-H_2O	b_{42}-NH_3	a_{46}-NH_3	y_{28}		
b_{9}	a_{16}-NH_3	y_{15}-NH_3	b_{43}	a_{47}-NH_3	y_{28}-H_2O		
b_{9}-H_2O	a_{24}-NH_3	y_{17}-NH_3	b_{45}-H_2O	a_{50}	y_{28}-NH_3		
b_{10}	a_{26}-NH_3	y_{22}	b_{45}-NH_3	a_{55}	y_{30}		
b_{11}	a_{27}-NH_3	y_{23}	b_{46}	a_{56}-NH_3	y_{31}		
b_{11}-H_2O	a_{29}	y_{24}	b_{52}		y_{31}-H_2O		
b_{11}-NH_3	a_{30}-NH_3	y_{24}-NH_3	b_{53}-NH_3		y_{31}-NH_3		
b_{13}-H_2O		y_{25}-NH_3	b_{53}-H_2O		y_{39}		
b_{13}-NH_3		y_{25}-H_2O	b_{49}		y_{39}-H_2O		
b_{15}-H_2O		y_{26}	b_{59}		y_{39}-NH_3		
b_{19}		y_{26}-H_2O	b_{59}-NH_3		y_{40}		
b_{21}		y_{26}-NH_3	b_{59}-H_2O		y_{42}		
b_{23}-H_2O			b_{60}		y_{42}-H_2O		
b_{23}-NH_3					y_{42}-NH_3		
b_{24}					y_{43}		
b_{25}					y_{45}		
b_{25}-H_2O					y_{46}-H_2O		
b_{25}-NH_3					y_{46}-NH_3		
b_{27}					y_{47}		
b_{29}-H_2O					y_{49}		
b_{29}-NH_3					y_{49}-H_2O		
b_{31}-H_2O					y_{49}-NH_3		
b_{31}-NH_3					y_{51}		
b_{32}					y_{52}		
b_{36}					y_{54}		
b_{36}-H_2O					y_{55}		
b_{36}-NH_3							

Table S3. Assigned fragments of 1451 m/z precursor after the MS/MS analysis of 1MEY# in the presence of 12.5 eq EDTA

ATCUN		C-term	1st ZF		3rd ZF		1st and 2nd ZF	2nd and 3rd ZF	other
	Ni^{2+}			Zn^{2+}		Zn^{2+}	Zn^{2+}	Zn^{2+}	Zn^{2+}
b_7	b_7+Ni^{2+} [U]	y_{26}	$b_{42}+Ni^{2+}$ [U]	-	y_{30} [U]	$y_{31}+Zn^{2+}$ [U]	$a_{83}+Zn^{2+}$ [U]	$y_{58}+Zn^{2+}$	$y_{98}+Zn^{2+}$
b_8	b_8+Ni^{2+}	y_{15}	$b_{43}+Ni^{2+}$		y_{31}	$y_{42}+Zn^{2+}$	$b_{86}+Zn^{2+}+Ni^{2+}$	$y_{58}-NH_3+Zn^{2+}$	$y_{98}-NH_3+Zn^{2+}$
b_9 [U]	a_8+Ni^{2+}	$y_{15}-NH_3$	$b_{46}+Ni^{2+}$		y_{42}	$y_{42}-H_2O+Zn^{2+}$	$a_{90}+Zn^{2+}$ [U]	$y_{59}+Zn^{2+}$ [U]	$y_{98}-H_2O+Zn^{2+}$
b_{11}	$b_{15}+Ni^{2+}$	$y_{15}-H_2O$	$b_{59}+Ni^{2+}$ [U]		$y_{42}-H_2O$	$y_{54}+Zn^{2+}$ [U]		$y_{82}+Zn^{2+}$ [U]	$y_{96}+Zn^{2+}$
b_{18}	$b_{18}+Ni^{2+}$	y_8-H_2O			y_{49}	$y_{30}+Zn^{2+}$			$y_{90}+Zn^{2+}$
	$b_{10}+Ni^{2+}$				y_{54}				$y_{86}+Zn^{2+}$
									$y_{86}-NH_3+Zn^{2+}$
									$y_{86}-H_2O+Zn^{2+}$
									$a_{98}-NH_3+Zn^{2+}+Ni^{2+}$ [U]
									$a_{98}+Zn^{2+}$

[U] indicates the uncertain fit of the fragments due to overlapping peaks. The assignment was performed manually in FreeStyle 1.6 (Thermo Scientific)

FIGURES

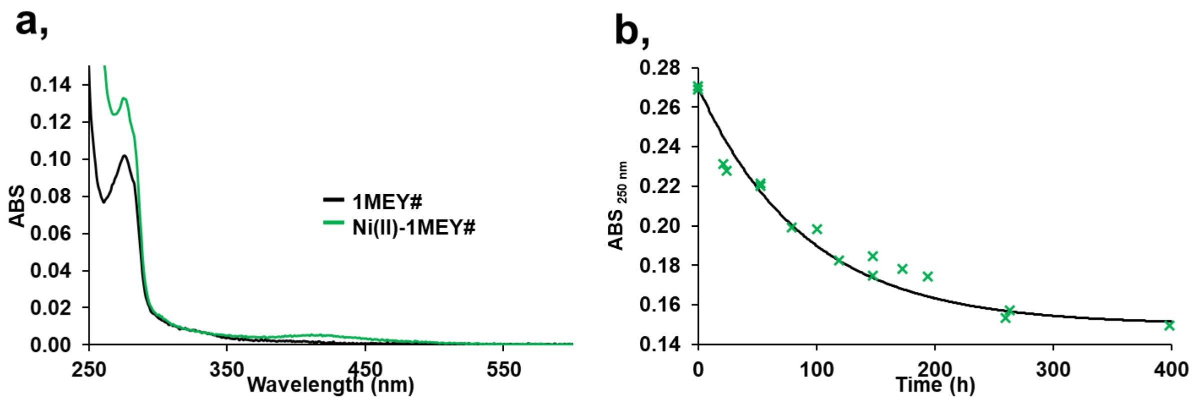

Figure S1. **a,** UV-Vis absorption spectra of 1MEY# in the presence (green line) and absence (black) of Ni(II) in the N-terminal ATCUN motif. **b,** Time dependent removal of Ni(II) from the N-terminal ATCUN motif of 1MEY# using 66 equivalents of EDTA as monitored at 250 nm by UV absorption spectrometry. c(1MEY#) = 25 µM in 10 mM HEPES (pH 7.4); *l* = 1 cm.

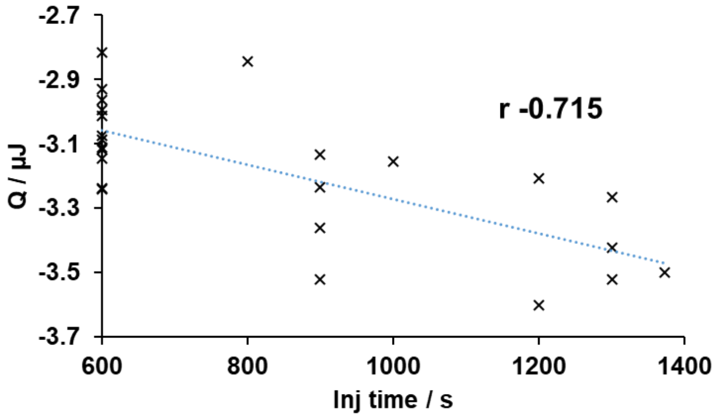

Figure S2. Impact of time between two subsequent injections on the measured heat in ITC experiments. These data were obtained from multiple EDTA–Zn(II) titrations. The data showed a strong negative Pearson correlation (r = –0.715), which justified the significance of this effect.

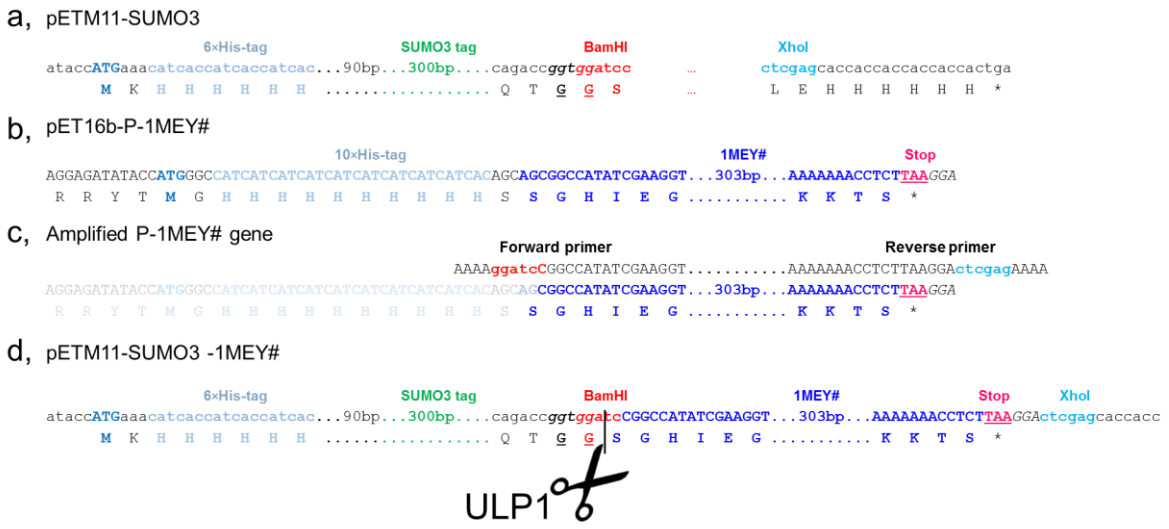

Figure S3. Construction of the pETM11-SUMO3-1MEY# plasmid. **a,** The cloning region of the new host plasmid pETM11-SUMO3. **b,** The pET16b-P-1MEY# plasmid used in our previous study. **c,** The oligonucleotide primers used for the amplification of the 1MEY# gene by polymerase chain reaction. **d,** The cloning region of the pETM11-SUMO3-1MEY# construct showing the cleavage site of the ULP1 protease.

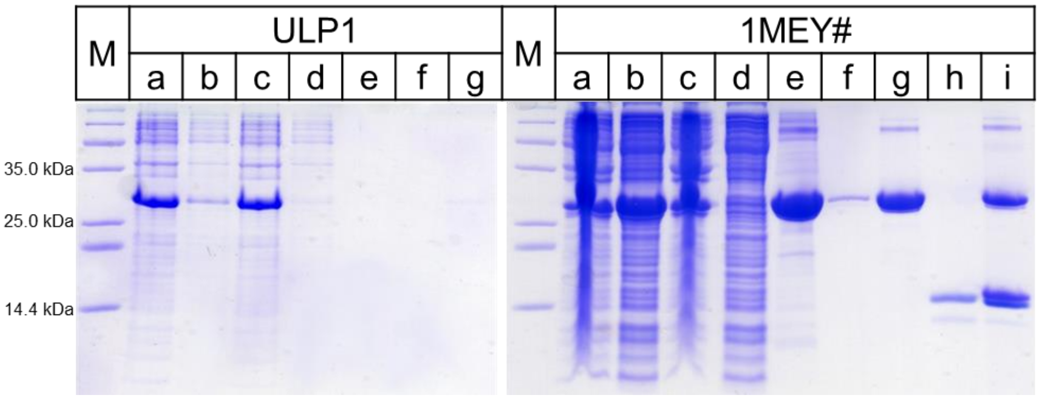

Figure S4. Monitoring of the Ni(II)-affinity batch purification procedure by SDS PAGE. The following explanations are effective for both the ULP1 (left) and 1MEY# (right) gel images. M: 5 µl Thermo Scientific Unstained Protein Molecular Weight Marker. Lane a: 10 µl aliquot of the total protein fraction obtained after the pellet was resuspended in 10 mL 1× binding buffer. Lane b: 10 µl aliquot of the soluble protein fraction. Lane c: 10 µl aliquot of the insoluble protein fraction. Lane d: 10 µl aliquot of the supernatant after mixing with the Ni(II)-loaded His•Bind resin. Lane e: 1 µl aliquot of the resin after protein binding. Lane f: 10 µl aliquot of the supernatant after washing with 6th Wash solution portion. Lane g: 1 µl aliquot of the resin after washing. Lanes h and i only appear in the gel image of 1MEY#. Lane h: 10 µl aliquot of the supernatant after ULP1 cleavage. Lane i: 1 µl aliquot of the resin after ULP1 cleavage. Based on the SDS-PAGE analysis ULP1 is extremely poorly soluble in the reaction buffers, but that small amount – hardly visible on the gel (lanes e, f, and g) – could cleave ~50% of the hexa-His-SUMO3 affinity tag from 1MEY# during 12 h incubation (lane i). ~50% of the cleaved 1MEY# stuck to the resin as it was observed previously [29]. In general ~25% of the initial protein was purified in the supernatant as pure native 1MEY# ZFP (lane h). 60 mM imidazole concentration was optimal for batch affinity tag cleavage. This allowed for a small soluble fraction of hexa-His-SUMO3-1MEY#. Lane f shows that a small portion of affinity-tagged 1MEY# can be found in the wash fraction under these conditions.

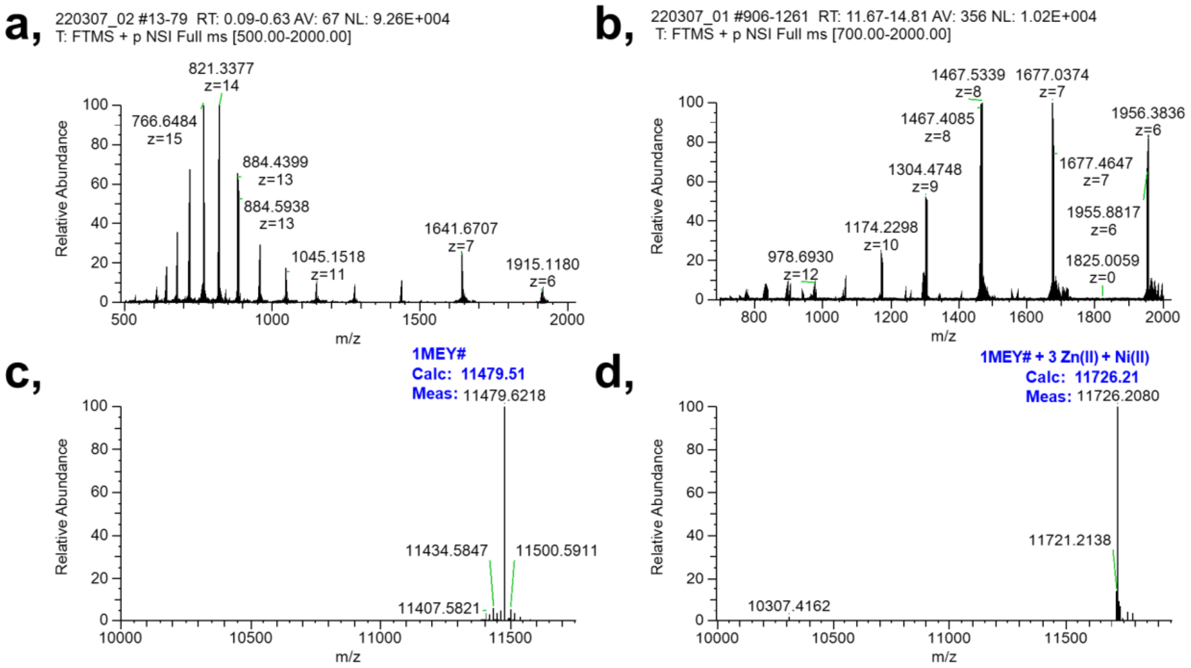

Figure S5. Mass spectra of the purified 1MEY#. **a,** The m/z spectrum of apo-protein. **b,** The m/z spectrum of holo-protein. **c,** Deconvoluted monoisotopic m/z spectrum of apo-protein (MH^+ ion); **d,** Deconvoluted monoisotopic m/z spectrum of holo-protein (MH^+ ion). No hexa-His-SUMO3 neither ULP1 fragment was detected in the mass spectra by analysing the major peaks

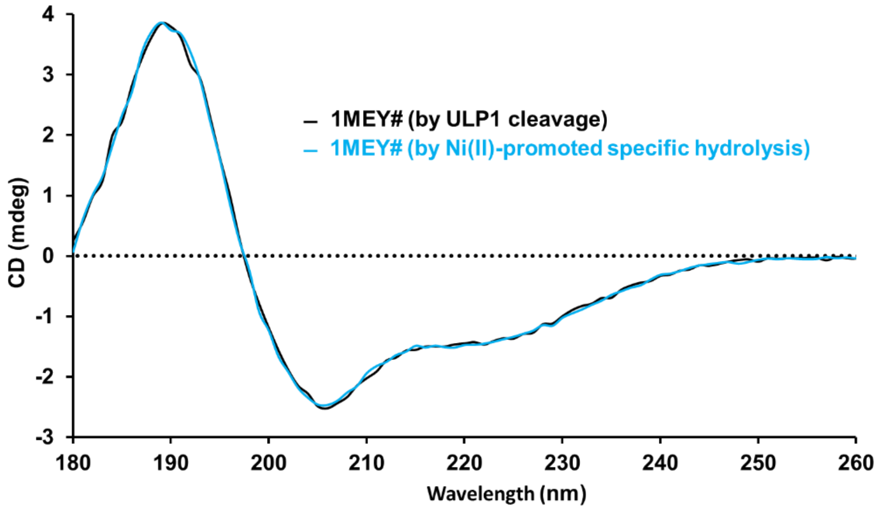

Figure S6. Comparison of the CD spectra of holo 1MEY# ZFPs obtained by Ni(II)-promoted specific hydrolysis of deca-His-1MEY# and by ULP1 cleavage of hexa-His-SUMO3-1MEY#. Protein concentrations were normalized to 18.8 μM (10 mM HEPES (pH 7.4) *l* = 0.1 mm).

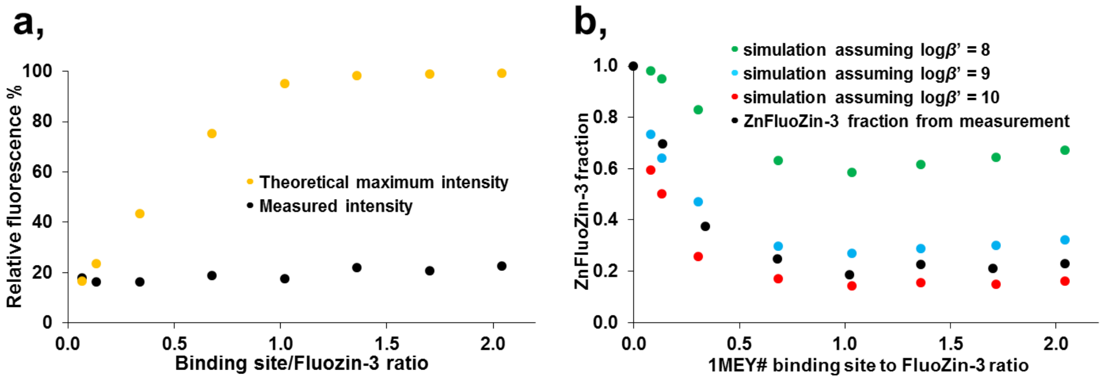

Figure S7. **a,** The fluorescence of the ZnFluoZin-3 complex in the presence of increasing amounts of holo-1MEY# (black dots). Reference measurements were also performed in parallel, in which equal amount of $ZnCl_2$ instead of the holo-1MEY#, was added to FluoZin-3 (yellow dots) yielding in high fluorescence intensity for comparison. The separately assembled samples (final concentrations: c(FluoZin-3) = 1.1 μM, c(holo-1MEY#) = 0 – 0.75 μM in 10 mM HEPES (pH 7.4); final volume: 150 μl) were incubated for 12 h at room temperature and then the fluorescence was recorded. **b,** The percentage of the ZnFluoZin-3 complex in the presence of increasing amounts of holo-1MEY# (black dots) achieved by dividing the measured intensity values (black dots Fig. S7. a,) with the theoretical maximum intensity values (yellow dots Fig. S7. a,)). Comparison with simulated data was performed assuming that $\log\beta'_{pH=7.4}$ of the Zn1MEY# complex is either 8 (green dots), 9 (light blue dots) or 10 (red dots). The simulations were performed by the PSEQUAD program [7].

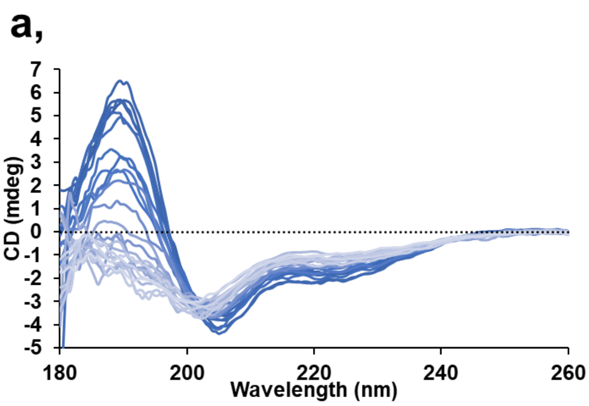

Figure S8. a, CD spectra of Zn(II)-loaded 1MEY# in the presence of increasing eqs EDTA. **b,** 190 nm ellipticity values versus EDTA to 1MEY# ratio. c(holo-1MEY#) = 16.4 µM, 10 mM HEPES (pH 8.2), *l* = 0.2 mm pathlength. Each sample was assembled separately and incubated for 5 min at 25 °C prior measurement.

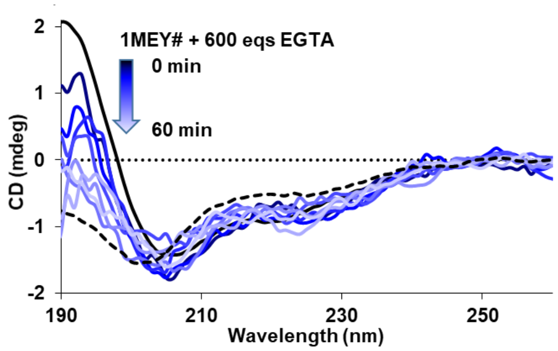

Figure S9. Time-dependence of 1MEY# CD spectra in the presence of 600 eqs EGTA. Black dashed line: 1MEY# in the presence of 5 eqs of EDTA after 5 min incubation (c(holo-1MEY#) = 9.3 µM in 10 mM HEPES (pH 7.4); *l* = 0.1 mm)

a,

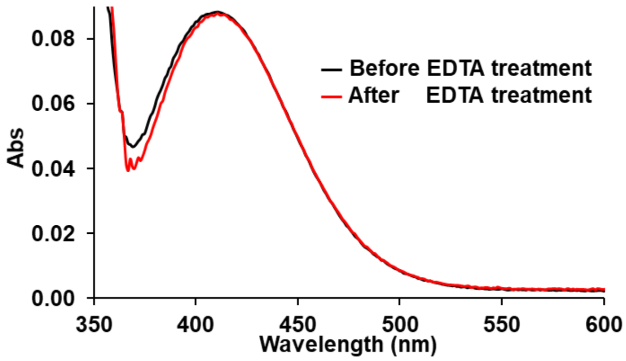

b,

	Before	After
$A_{412\ nm}$	0.0883	0.0874
$c_{Cysteines}$ (μM)	6.24	6.18

Figure S10. a, DTNB assay of 1MEY# ZFP before and after 200 equiv EDTA treatment. EDTA treatment conditions: 20 μL 20 μM Zn(II)-loaded 1MEY#, 400 μM EDTA, 60 min, 25 °C incubation in 10 mM HEPES (pH 7.4). Pre assay treatment: 20 μL 20 μM Zn(II)-loaded 1MEY# + 5 μL 10 w/v % SDS + 2 μL 40 mM EDTA. Assay conditions: 400 μL 1 μM 1MEY# ZFP, 65 μg/ml DTNB, 100 mM phosphate buffer (pH 7.2) 5 min, 25 °C incubation. **b,** Determined absorbance at 412 nm and the determined cysteine concentrations inside the measured samples.

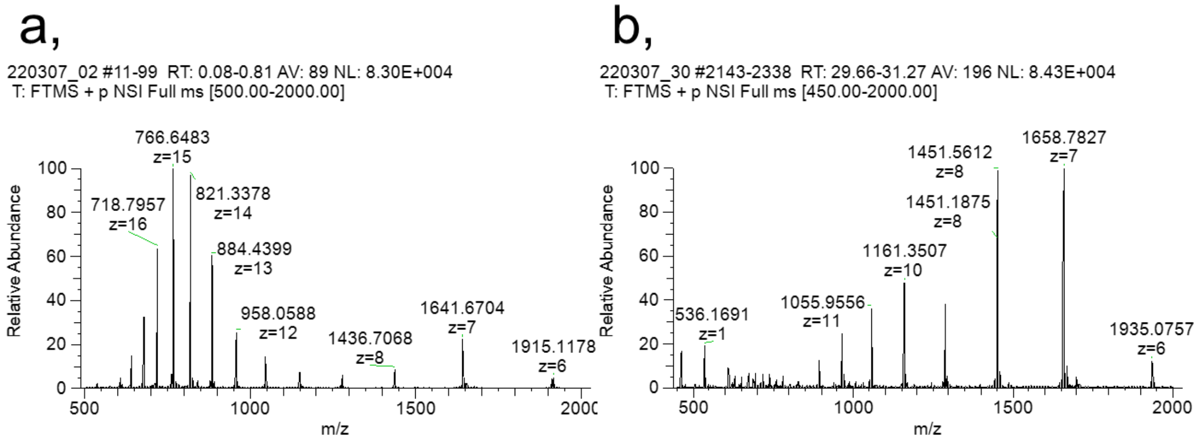

Figure S11. Mass spectra of **a,** apo-1MEY#; **b,** holo-1MEY# in the presence of 12.5 eqs. of EDTA. In panel **a,** all peaks with higher than 5% relative abundance were assigned. These peaks were all related to apo-1MEY# in multiple charge states. In panel **b,** all peaks with higher than 10% relative abundance were assigned. These peaks were all related to Ni(II)$_1$Zn(II)$_2$1MEY# and Ni(II)$_1$Zn(II)$_1$1MEY#. “z” indicates the charge. Multiple peaks could be seen because these MS spectra were not deconvoluted.

REFERENCES

[1] Hay R W, Hassan M M, You-Quan C (1993) Kinetic and thermodynamic studies of the copper(II) and nickel(II) complexes of glycylglycyl-L-histidine. J Inorg Biochem 52:17-25 https://doi.org/10.1016/0162-0134(93)85619-j

[2] Belczyk-Ciesielska A, Csipak B, Hajdu B, Sparavier A, Asaka M N, Nagata K, Gyurcsik B, Bal W (2018) Nickel(II)-promoted specific hydrolysis of zinc finger proteins. Metallomics 10:1089-1098 https://doi.org/10.1039/c8mt00098k

[3] Sambrook J, Russel D W (2006) Purification of nucleic acids by extraction with phenol:chloroform in Cold Spring Harbor Protocols https://doi.org/10.1101/pdb.prot4455

[4] Malakhov M P, Mattern M R, Malakhova O A, Drinker M, Weeks S D, Butt T R (2004) SUMO fusions and SUMO-specific protease for efficient expression and purification of proteins. J Struct Funct Genomics 5:75–86 https://doi.org/10.1023/B:JSFG.0000029237.70316.52

[5] Gerhardt P, Murray R G, Krieg N R, Wood W A (1994) Methods for General and Molecular Bacteriology American Society for Microbiology Washington DC

[6] Anderegg G (1977) Critical Survey of Stability Constants of EDTA Complexes In: Critical Evaluation of Equilibrium Constants in Solution: Stability Constants of Metal Complexes 1st edn. Pergamon https://doi.org/10.1016/C2013-0-02924-6

[7] Zékány L, Nagypál I (1985) PSEQUAD. In: Leggett D J (ed) Computational Methods for the Determination of Formation Constants. Modern Inorganic Chemistry Springer, Boston https://doi.org/10.1007/978-1-4684-4934-1_8

[8] Marini M A, Evans W J, Berger R L (1985) Use of the twin-cell differential titration calorimeter for binding studies. I. EDTA and its calcium complex. J Biochem Bioph Meth 10:273-285 https://doi.org/10.1016/0165-022X(85)90061-2

[9] Fukada H, Takahashi K (1998) Enthalpy and heat capacity changes for the proton dissociation of various buffer components in 0.1 M potassium chloride. Proteins: Struct Funct Bioinf 33:159-166 https://doi.org/10.1002/(SICI)1097-0134(19981101)33:2<159::AID-PROT2>3.0.CO;2-E

[10] Alderighi L, Lucia P, Midollini S, Vacca A (2003) Coordination chemistry of the methylmercury(II) ion in aqueous solution: a thermodynamic investigation. Inorg Chim Acta 356:8-18 https://doi.org/10.1016/S0020-1693(03)00317-7

[11] Blasie C A, Berg J M (2002) Structure-based thermodynamic analysis of a coupled metal binding-protein folding reaction involving a zinc finger peptide. Biochemistry 41:15068-15073 https://doi.org/10.1021/bi026621h

[12] Sénèque O, Latour J-M (2010) Coordination Properties of Zinc Finger Peptides Revisited: Ligand Competition Studies Reveal Higher Affinities for Zinc and Cobalt. J Am Chem Soc 132:17760-17774 https://doi.org/10.1021/ja104992h

[13] Webster K A, Prentice H, Bishopric N H (2001) Oxidation of Zinc Finger Transcriprion Factors: Physiological Consequences. Antioxid Redox Signal 3:535-548 https://doi.org/10.1089/15230860152542916

[14] Kluska K, Adamczyk J, Krężel A (2018) Metal binding properties stability and reactivity of zinc fingers. Coord Chem Rev 367:18-64 https://doi.org/10.1016/j.ccr.2018.04.009

[15] Bjerrum J (1941) Metal amine formation in aqueous solution. Haase, Copenhagen

[16] Beck M I, Nagypál I (1990) Chemistry of complex equilibria. Akadémiai Kiadó and Ellis Horwood Limited Publishers, Budapest and Chichester

[17] Krizek B A, Amann B T, Kilfoil V J, Merkle D L, Berg J M (1991) A consensus zinc finger peptide: design high-affinity metal binding a pH-dependent structure and a His to Cys sequence variant. J Am Chem Soc 113:4518–4523 https://doi.org/10.1021/ja00012a021

[18] Krizek B A, Merkle D L, Berg J M (1993) Ligand variation and metal ion binding specificity in zinc finger peptides. Inorg Chem 32:937-940 https://doi.org/10.1021/ic00058a030

[19] Kluska K, Chorążewska A, Peris-Díaz M D, Adamczyk J, Krężel A (2022) Non-Conserved Amino Acid Residues Modulate the Thermodynamics of Zn(II) Binding to Classical betabetaalpha Zinc Finger Domains. Int J Mol Sci 23:1-17 https://doi.org/10.3390/ijms232314602

[20] Berg J M , Merkle D L (1989) On the metal ion specificity of zinc finger proteins. J Am Chem Soc 111:3759-3761 https://doi.org/10.1021/ja00192a050

[21] Miłoch A, Krężel A (2014) Metal binding properties of the zinc finger metallome-insights into variations in stability. Metallomics 6:2015-2024 https://doi.org/10.1039/C4MT00149D

[22] Kocyła A, Pomorski A, Krężel A (2015) Molar absorption coefficients and stability constants of metal complexes of 4-(2-pyridylazo)resorcinol (PAR): Revisiting common chelating probe for the study of metalloproteins. J Inorg Biochem 152:82-92 https://doi.org/10.1016/j.jinorgbio.2015.08.024

23 Guerrerio A L, Berg J M (2004) Metal Ion Affinities of the Zinc Finger Domains of the Metal Responsive Element-Binding Transcription Factor-1 (MTF1)†. Biochemistry 43:5437–5444 https://doi.org/10.1021/bi0358418

[24] Bulathge A W, Villones R L E, Herbert F C, Gassensmith J J, Meloni G (2022) Comparative cisplatin reactivity towards human Zn7-metallothionein-2 and MTF-1 zinc fingers: potential implications in anticancer drug resistance. Metallomics 14:1-18 https://doi.org/10.1093/mtomcs/mfac061

[25] Lachenmann M J, Ladbury J E, Qian X, Huang K, Singh R, Weiss M A (2009) Solvation and the hidden thermodynamics of a zinc finger probed by nonstandard repair of a protein crevice. Protein Sci 13:3115-3126 https://doi.org/10.1110/ps.04866404

[26] Chan K L, Bakman I, Marts A R, Batir Y, Dowd T L, Tierney D L, Gibney B R (2014) Characterization of the Zn(II) Binding Properties of the Human Wilms' Tumor Suppressor Protein C-terminal Zinc Finger Peptide. Inorg Chem 53:6309-6320 https://doi.org/10.1021/ic500862b

[27] Posewitz M C, Wilcox D E (1995) Properties of the Sp1 Zinc Finger 3 Peptide: Coordination Chemistry Redox Reactions and Metal Binding Competition with Metallothionein. Chem Res Toxicol 8:1020-1028 https://doi.org/10.1021/tx00050a005

[28] Rich A M, Bombarda E, Schenk A D, Lee P E, Cox E H, Spuches A M, Hudson L D, Kieffer B, Wilcox D E (2012) Thermodynamics of Zn2+ Binding to Cys2His2 and Cys2HisCys Zinc Fingers and a Cys4 Transcription Factor Site. J Am Chem Soc 134:10405-10418 https://doi.org/10.1021/ja211417g

[29] Abd Elhameed HAH, Hajdu B, Balogh R K, Hermann E, Hunyadi-Gulyás É, Gyurcsik B (2019) Purification of proteins with native terminal sequences using a Ni(II)-cleavable C-terminal hexahistidine affinity tag. Protein Expr Purif 159:53-59 https://doi.org/10.1016/j.pep.2019.03.009
